# Graft-versus-host disease reduces lymph node display of tissue-restricted self-antigens and promotes autoimmunity

**DOI:** 10.1172/JCI133102

**Published:** 2020-03-09

**Authors:** Simone Dertschnig, Pamela Evans, Pedro Santos e Sousa, Teresa Manzo, Ivana R. Ferrer, Hans J. Stauss, Clare L. Bennett, Ronjon Chakraverty

**Affiliations:** 1 UCL Cancer Institute, and; 2 Institute of Immunity and Transplantation, London, United Kingdom.; 3 European Institute of Oncology, Milan, Italy.

**Keywords:** Autoimmunity, Transplantation, Bone marrow transplantation, Stem cell transplantation, Tolerance

## Abstract

Acute graft-versus-host disease (GVHD) is initially triggered by alloreactive T cells, which damage peripheral tissues and lymphoid organs. Subsequent transition to chronic GVHD involves the emergence of autoimmunity, although the underlying mechanisms driving this process are unclear. Here, we tested the hypothesis that acute GVHD blocks peripheral tolerance of autoreactive T cells by impairing lymph node (LN) display of peripheral tissue–restricted antigens (PTAs). At the initiation of GVHD, LN fibroblastic reticular cells (FRCs) rapidly reduced expression of genes regulated by DEAF1, an autoimmune regulator-like transcription factor required for intranodal expression of PTAs. Subsequently, GVHD led to the selective elimination of the FRC population, and blocked the repair pathways required for its regeneration. We used a transgenic mouse model to show that the loss of presentation of an intestinal PTA by FRCs during GVHD resulted in the activation of autoaggressive T cells and gut injury. Finally, we show that FRCs normally expressed a unique PTA gene signature that was highly enriched for genes expressed in the target organs affected by chronic GVHD. In conclusion, acute GVHD damages and prevents repair of the FRC network, thus disabling an essential platform for purging autoreactive T cells from the repertoire.

## Graphical abstract

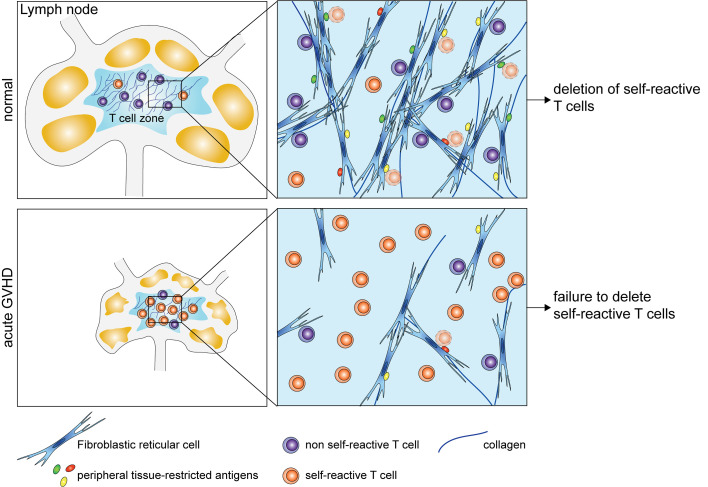

## Introduction

The success of allogeneic hematopoietic stem cell transplantation is limited by the occurrence of graft-versus-host disease (GVHD), an acute inflammatory process triggered by the influx of alloreactive effector T cells into barrier surface tissues (primarily skin and gut) and lymphoid organs (1). Although acute GVHD may resolve with corticosteroid treatment, its occurrence is a strong predictor for the later development of chronic GVHD; in this case, organ involvement is often more extensive than the acute disease and clinical features include many features reminiscent of classic autoimmune disorders (e.g., scleroderma, sicca syndrome, and immune cytopenias) (2).

The mechanisms underpinning the transition from acute to chronic GVHD are poorly understood. Donor T cells with autoreactivity can readily be detected 2–5 weeks after the onset of acute GVHD in mice (3–6); autoreactivity is inferred because these T cells can induce widespread tissue injury upon secondary transfer to recipient mice syngeneic to the donor and do so with patterns that recapitulate those observed in chronic GVHD (4, 6, 7). Acute GVHD also targets the thymus and disrupts central tolerance, the process by which T cells reactive against self-antigens are eliminated from the repertoire (5, 8–11). Medullary thymic epithelial cells (mTECs) are particularly sensitive to immune injury in GVHD (9); these stromal cells display peripheral tissue–restricted antigens (PTAs) through a process that requires the transcription factor autoimmune regulator (AIRE) and are normally required for the negative selection of self-reactive thymocytes (12). Loss of mTECs in mice with acute GVHD therefore allows escape of autoreactive T cells into the periphery (8). A 2-hit hypothesis for the development of chronic GVHD has been proposed that invokes this loss of central tolerance (hit 1) and a second insult to peripheral tolerance mechanisms (hit 2), creating conditions favoring unchecked T cell autoreactivity and inflammation (8, 13). A better understanding of how such peripheral regulatory mechanisms fail in GVHD will be critical to preventing the emergence of autoimmunity and chronic tissue injury.

Similar to mTECs, lymph node (LN) nonhematopoietic stromal cells directly present PTAs and also trigger deletion of self-reactive T cells (14–17). PTA display may exist to reinforce tolerance of autoreactive T cells escaping thymic negative selection or alternatively, provide a means of purging the repertoire of T cells directed to self-antigens not expressed by mTECs. Individual LN stromal populations express distinct repertoires of PTAs regulated by mechanisms that are AIRE independent, for example involving the AIRE-like transcription factor, deformed epidermal autoregulatory factor 1 homolog (DEAF1) (14, 18). The fibroblastic reticular cell (FRC) population is a subset of LN stromal cells lacking hematopoietic (CD45) and endothelial (CD31) markers but expressing a small membrane glycoprotein, podoplanin (or gp38), and the constitutive chemokine CCL19; they form a physical scaffold within the T cell zone and are particularly well positioned to present PTAs to naive T cells in the steady state (19).

In contrast to their inhibitory functions in regulating T cell autoreactivity to PTAs, FRCs have recently been shown to initiate T cell reactivity to alloantigens in GVHD (20). This role in priming depends on upregulated expression of Notch ligands by FRCs and may explain the subsequent targeting of this population by the ensuing alloreactive T cell response, leading eventually to severe disruption of the FRC network in several models of GVHD (21). Damage to the FRC network and overall LN structure in murine GVHD mirrors the damage to the T cell zones of LNs described in human patients following transplant (22, 23). FRC targeting in GVHD correlates with profound defects in T cell–dependent antibody immunity (21), a finding consistent with known functions of FRCs in promoting cell interactions and LN remodeling to accommodate rapidly expanding immune populations (24, 25).

FRC network injury can also occur following viral infection (e.g., acute lymphocytic choriomeningitis, LCMV) but is followed by rapid restoration upon clearance of infection (26). Repair of the FRC network following LCMV infection triggers a lymphoid organ transcriptional reorganization program involving increased expression of several genes (e.g., *Vcam1*, *Icam1*, *Cxcl13*, and *Ltbr*) that are essential for the formation of LNs in the embryo and critical for the crosstalk between lymphoid tissue organizer (LTo) cells (putative precursors to the FRC population) and lymphoid tissue inducer (LTi) cells that express an isoform of RAR-related orphan receptor gamma (RORγt). Scandella and colleagues have proposed that FRC injury in acute LCMV infection in adults recapitulates the embryonic process, where emergence of a reorganizational transcriptional signature is accompanied by rapid LN accumulation of RORγt^+^ LTi cells (26). Lack of RORγt^+^ LTi-like cells at the time of acute LCMV infection was shown to impair FRC network restoration (26). Whether such a repair process is operative in the context of FRC injury in GVHD is not known.

Although disruption to the FRC network of LNs may help to explain the characteristic immune deficiency of GVHD, its impact on peripheral tolerance has not been examined to our knowledge. Here, we tested the hypothesis that degeneration of LN stroma during acute GVHD disrupts their role in the peripheral education of self-reactive T cells. We find that the FRC network fails to regenerate following the onset of acute GVHD even when the initial immune response is curtailed. As a consequence of early PTA gene downregulation and subsequent loss of the FRC network, the normal process of purging of autoaggressive CD8^+^ T cells from the peripheral repertoire does not occur. Finally, we show that steady-state FRCs express a distinct PTA gene signature that is highly enriched for genes normally expressed in target organs affected by chronic GVHD. Thus, repair of stromal populations in lymphoid organs and restoration of PTA display may be essential to prevent the transition from acute to chronic GVHD.

## Results

### Acute transcriptional response of FRCs to GVHD.

**Figure 1:**
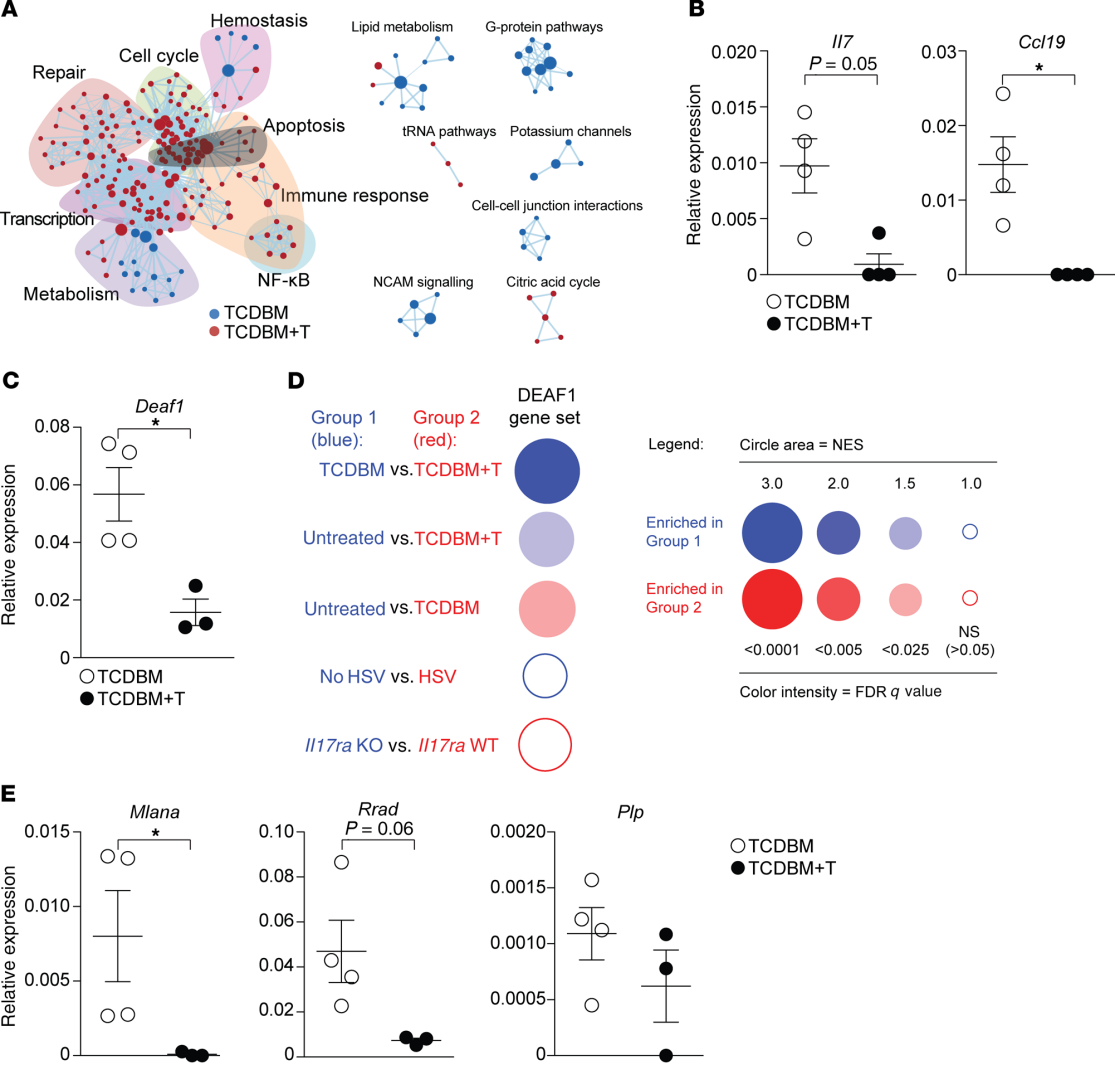
Acute transcriptional response of FRCs to GVHD. (**A**) Network visualization of differentially upregulated REACTOME pathways in FRCs at day 7 after allo-BMT using EnrichmentMap. Enriched REACTOME pathways are depicted by red and blue nodes, where blue represents significant upregulation in TCDBM versus TCDBM+T and red represents significant upregulation in TCDBM+T versus TCDBM. (**B**) FRC populations were flow sorted from recipients with or without acute GVHD and expression of *Il7* and *Ccl19* was analyzed by quantitative RT-PCR (qPCR). Expression of the gene of interest is shown relative to the expression of the housekeeping gene *Gapdh*. (**C**) Expression of *Deaf1* in sorted FRCs by qPCR. Expression of the gene of interest is shown relative to the expression of the housekeeping gene *Gapdh*. (**D**) Expression of DEAF1-dependent genes was analyzed by GSEA of RNA-seq data derived from FRCs isolated from GVHD^+^ (TCDBM, blue) versus GVHD^–^ (TCDBM+T, red) recipients, from HSV-infected versus control noninfected mice, or from vaccinated mice in which FRCs selectively lacked IL-17Rα versus wild-type controls. Results are represented in a BubbleGUM plot in which stronger and more significant enrichments are represented by larger and darker bubbles. Blue and red colors indicate enrichment in group 1 or group 2, respectively, as indicated by the text to the left of each bubble. (**E**) Expression of known FRC-specific PTA genes relative to *Gapdh* was analyzed by qPCR in sorted FRCs from mice with or without GVHD by qPCR. Data represent mean ± SEM. *P < 0.05 by Mann-Whitney *U* test.

To permit rapid remodelling of the LN, FRCs show exquisite sensitivity to a broad spectrum of proinflammatory stimuli, modulating expression of genes with functions relating to the cell cycle and survival (27–29). To determine the acute transcriptional response of FRCs to inflammation induced by GVHD, we performed RNA sequencing (RNA-seq) analysis of FRCs (identified as a CD45^–^gp38^+^CD31^–^ population) isolated from mice with and without GVHD on day 7. In these experiments, GVHD was induced following an MHC-matched (B6, H-2^b^), female→male (F→M) bone marrow transplantation (BMT) by cotransfer of T cell–depleted bone marrow (TCDBM) and CD8^+^ MataHari (Mh) T cells transgenic for a T cell receptor (TCR) reactive with male antigen (TCDBM+T) (30). Compared with no-GVHD controls (TCDBM), FRCs isolated from GVHD mice (TCDBM+T) showed increased representation of gene ontology (GO) terms for cell cycle, apoptosis, NF-κB activation, and DNA repair. In contrast, we observed reduced representation of GO terms associated with cell morphogenesis, including those relating to formation of branching structures and vascularization (Figure 1A). Although there were some differences (for example, increased expression of pathways relating to NF-κB activation in acute GVHD), we observed remarkably similar changes in gene expression in FRCs early following herpes simplex virus (HSV) infection (27), suggesting that components of the transcriptional response represent default programs triggered by inflammation (Supplemental Figure 1; supplemental material available online with this article; https://doi.org/10.1172/JCI133102DS1). However, in sharp contrast to other inflammatory conditions where FRC population expansion is induced (27–29), we observed (using quantitative real-time PCR [RT-PCR]) early downregulation of the genes *Il7* and *Ccl19*, genes that are critical for FRC functions in supporting the survival and homing of naive T cells (19) (Figure 1B). We also sought to determine how the acute transcriptional response to GVHD would affect PTA gene expression in FRCs. We first examined expression of genes encoding AIRE and DEAF1, transcriptional regulators of PTA expression in the thymus (31) and LNs (18), respectively. Consistent with published data (15), *Aire* gene expression was not detectable in FRCs under any condition (data not shown). *Deaf1* was expressed in control FRCs, as described previously (15), but its expression was significantly reduced in the presence of acute GVHD (Figure 1C). To determine if expression levels of genes regulated by DEAF1 were also reduced in GVHD, we used gene set enrichment analysis (GSEA) to determine enrichment or otherwise of 157 DEAF1-dependent genes (defined as genes with ≥ 3-fold reduced expression in LN stromal cells from *Deaf1-*knockout versus wild-type mice) (18). As shown in Figure 1D, GVHD was associated with downregulation of this gene set in FRCs (normalized enrichment score [NES] –2.45, FDR *q* value = 0.0007 for TCDBM+T versus TCDBM comparison). Downregulation of DEAF1-dependent genes in FRCs was specific to GVHD and not generalizable to BMT alone, or to LN FRC responses to other inflammatory stimuli, including to HSV infection (27) or to IL-17 following vaccination (28). Finally, we used RT-PCR to evaluate how GVHD affected the expression of specific PTA genes known to be expressed by FRCs (*Mlana*, *Plp*, and *Rrad*) (15). As shown in Figure 1E, *Mlana* (encoding melan-A, expressed in skin) was significantly reduced, with a similar trend for *Rrad* (encoding ras-related glycolysis inhibitor and calcium channel regulator, expressed in muscle and lung) but not for *Plp* (encoding proteolipid 1, expressed in brain). Thus, FRCs show a complex acute transcriptional response to GVHD that includes early downregulation of genes critical to their core functions in supporting survival of naive T cells as well as their role in the display of PTAs.

### Damage to the FRC network following acute GVHD is irreversible.

**Figure 2:**
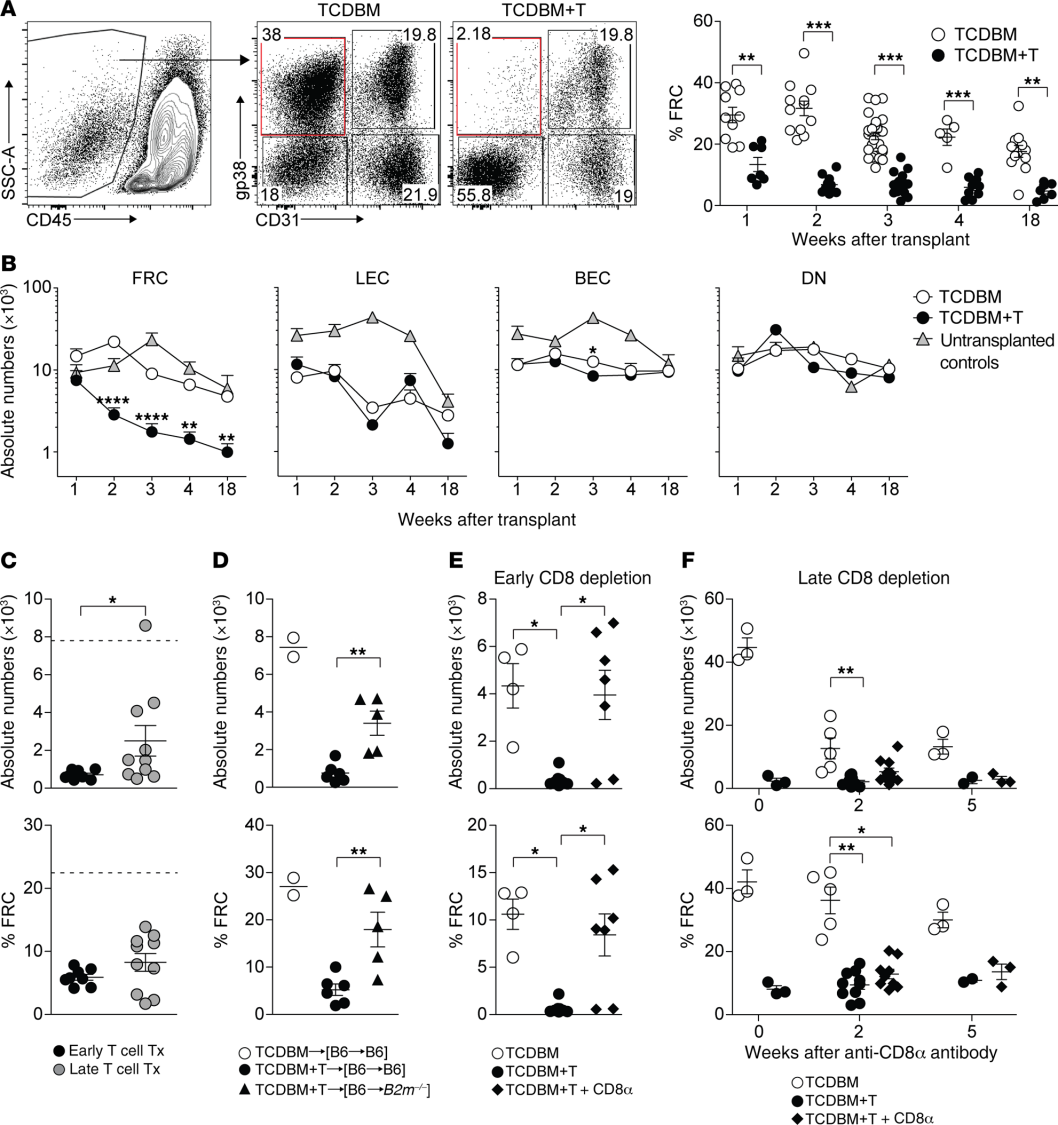
The extent and duration of FRC network injury affect its capacity for regeneration. (**A**) LN stromal cells were analyzed at indicated time points after allo-BMT using the gating strategy shown. Frequencies of FRCs among CD45^–^ LN stromal cells are shown at indicated time points after BMT in TCDBM and TCDBM+T recipients. (**B**) Absolute cell numbers of LN stromal cell subsets at indicated time points after F→M BMT with either TCDBM alone or TCDBM+T. Untransplanted, age-matched mice were used as controls (data derived from 10 independent experiments). (**C**) Absolute numbers (top) and frequencies (bottom) of FRCs at 3 weeks following transplantation in F→M BMT receiving Mh T cells either on day 0 (early) or 7 (late) after BMT. Dotted line indicates FRC numbers or frequencies measured in control recipients (data are representative of 3 independent experiments). (**D**) FRC frequencies and absolute numbers following second BMT and transfer of Mh T cells to [B6 male→*B2m^–/–^* male] versus [B6 male→B6 male] BM chimeras. Plots show absolute numbers (top) and frequencies (bottom) of FRCs among CD45^–^ LN stromal cells (data are representative of 2 independent experiments). (**E**) Absolute numbers (top) and frequencies (bottom) of FRCs in recipients that either received TCDBM alone or TCDBM+T with or without early anti-CD8α antibody given twice weekly. LN stromal cells were analyzed on day 28 after transplantation, (**F**) Absolute numbers (top) and frequencies (bottom) of FRCs in recipients that either received TCDBM alone or TCDBM+T with or without late (from day 14) anti-CD8α antibody to deplete donor CD8^+^ T cells. LN stromal cells were analyzed at indicated time points following the start of anti-CD8α treatment (data derived from 2 independent experiments). Data represent mean ± SEM. *P < 0.05; **P < 0.01; ***P < 0.001; ****P < 0.0001 by Mann-Whitney *U* test (**A** and **C**) or Kruskal-Wallis ANOVA (**B** and **D**–**F**).

Intranodal PTA display in GVHD will be affected not only by expression levels of relevant antigens by individual stromal cells but also on the overall integrity of each of the populations. To address how acute GVHD would affect peripheral LN (PLN) stroma overall, we tracked stromal numbers over time in the F→M BMT model. Using the gating strategy shown in Figure 2A, we found that FRC numbers progressively fell by approximately 10-fold following the onset of GVHD over several weeks with no evidence of recovery at 18 weeks; in contrast, the numbers of other major stromal populations, lymphatic endothelial cells (LECs) and blood endothelial cells (BECs), remained intact (Figure 2, A and B). Loss of FRCs was confirmed by confocal immunofluorescence imaging and associated with marked disruption of LN paracortex structure (Supplemental Figure 2A). The extent of FRC depletion (compared with baseline) was less if Mh T cells were transferred after a delay of 7 days, a situation where the severity of GVHD is significantly reduced (32), indicating that the degree of alloreactivity is important in dictating injury to this population (Figure 2C). To test if FRC targeting in this CD8^+^ T cell–dependent model required cognate interaction with MHC class I–expressing target cells, we established BM chimeras where radioresistant stromal cells either did or did not express MHC class I (i.e., [B6 male→B6 male] versus [B6 male→*B2m^–/–^* male] BM chimeras, respectively) and then, induced GVHD following a second BMT. As shown in Figure 2D, lack of MHC class I expression by stroma protected the FRC population from GVHD-induced injury. As previously demonstrated following acute LCMV infection (26), CD8^+^ T cell targeting of FRCs in acute GVHD was independent of the perforin pathway (Supplemental Figure 2B). However, MHC class I–restricted targeting of FRCs was not required for their elimination, as HY-specific CD4^+^ T cells could also induce FRC loss following F→M BMT, albeit the extent of injury was less than observed in the CD8^+^ T cell–dependent model (Supplemental Figure 2C). The long time frame afforded by the Mh model (survival is ~50% at 18 weeks in mice with GVHD) allowed us to determine if FRC regeneration could occur if acute GVHD was terminated early during its evolution. In the Ccl19.DTR model, administration of diphtheria toxin (DT) induces complete ablation of the FRC population, with partial recovery evident at 2 weeks and almost full recovery at 4 weeks (33). Thus, we sought to measure the long-term integrity of the FRC network under conditions where GVHD had been terminated at an earlier time point using anti-CD8α antibody depletion following BMT and T cell transfer (Supplemental Figure 2D shows the effect of anti-CD8α antibody on clinical GVHD; median CD8^+^ T cell numbers at 4 weeks were 4.23% of live gate in control versus 0.007% in antibody-treated mice, *P* < 0.0001, Mann-Whitney test, 2-tailed). If anti-CD8α antibody was given from day 5 after BMT (a time point when the majority of the FRC network remains intact; Figure 2B), we found that FRC numbers were preserved to a similar extent as controls without GVHD when evaluated at the 4-week time point (Figure 2E). We next asked whether the FRC network could recover if GVHD was terminated at the later time point of day 14 when substantial loss of the FRC population had already occurred (Figure 2B). As shown in Figure 2F, if the start of anti-CD8α antibody treatment was delayed to 14 days after BMT and T cell transfer, FRC numbers did not recover over the next 5 weeks in the CD8-depleted mice when compared with controls without GVHD. A slight trend toward an increase in FRC numbers in the CD8-depleted compared with the nondepleted group was observed at the 2-week time point, indicative of less exposure to the process causing immune injury and/or an abortive attempt at recovery, but this effect was only transient. Together, these data indicate that the capacity for the FRC network to remain intact is dependent on the extent and/or duration of injury induced by GVHD; the longer the duration of injury, the lower the capacity for FRC regeneration.

**Figure 3:**
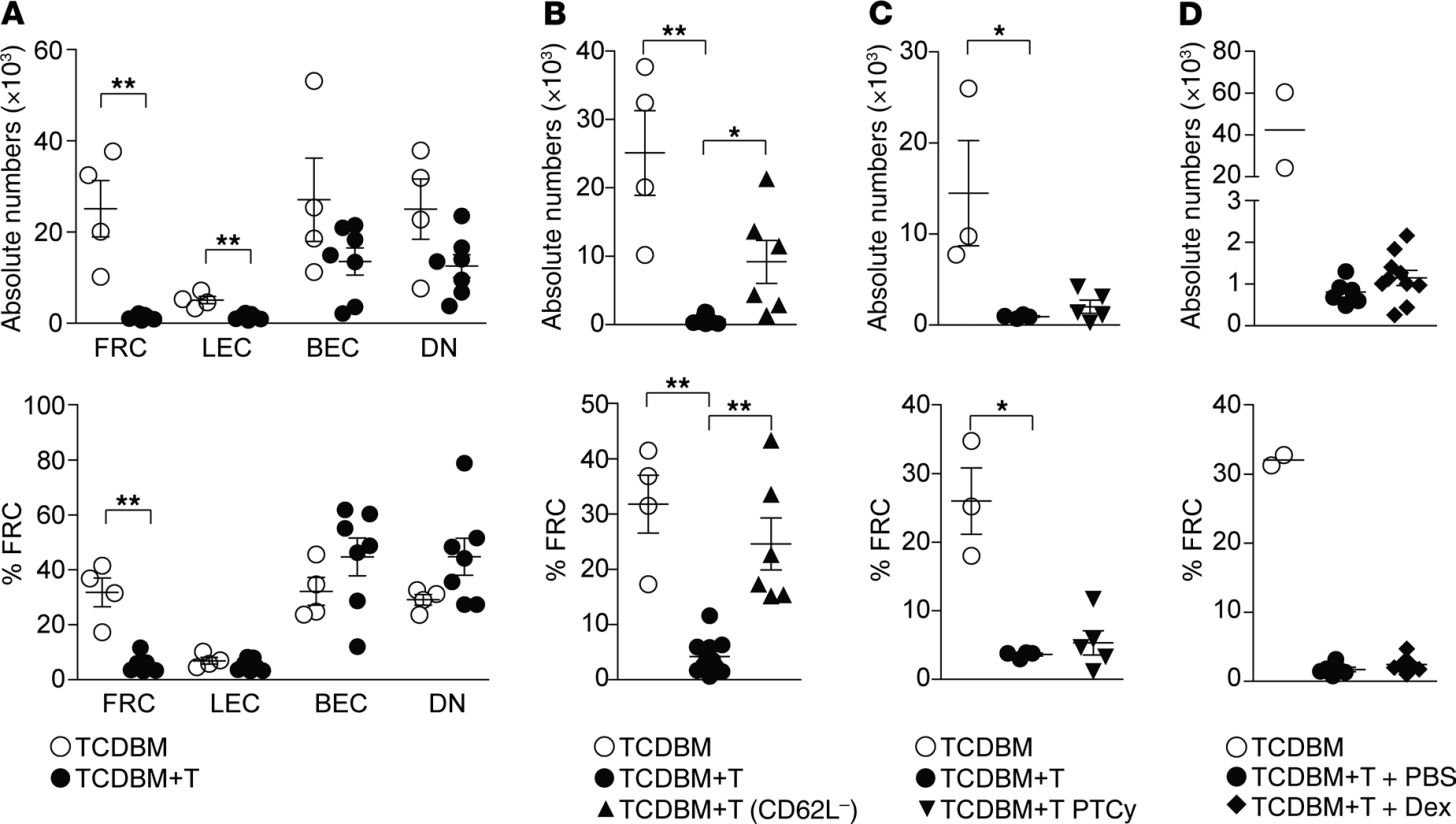
Effect of clinical strategies for GVHD prevention or treatment on FRC network integrity. (**A**) Absolute cell numbers (top) and frequencies (bottom) of LN stromal cell subsets in B6→129 model on day 21 after BMT (data derived from 3 independent experiments). (**B**) Absolute FRC numbers and frequencies 21 days following BMT with either TCDBM alone, TCDBM + polyclonal T (TCDBM+T), or TCDBM + CD62L^–^ T cells (data derived from 4 independent experiments). (**C**) Absolute FRC numbers (top) and frequencies (bottom) 18 days following BMT with either TCDBM alone or TCDBM+T with or without posttransplant cyclophosphamide (PTCy) treatment. PTCy was administered on days 3 and 4 after transplant (25 mg/kg/day). (**D**) Absolute FRC numbers (top) and frequencies (bottom) 19 days following BMT with TCDBM alone, or TCDBM+T treated with dexamethasone (0.3 mg/kg/day), or PBS starting on day 5 after BMT (data derived from 2 independent experiments). Data represent mean ± SEM. *P < 0.05; **P < 0.01 by Mann-Whitney *U* test (**A**) or Kruskal-Wallis ANOVA (**B** and **C**).

We next addressed how clinical strategies designed to prevent or treat GVHD in human patients would affect the integrity of the LN FRC population. In these experiments, we employed a clinically relevant MHC-matched (H-2^b^), multiple minor antigen–mismatched model of BMT (B6→129) where GVHD is more severe than the F→M model (survival 40%–50% at 3 weeks). We found that GVHD induced similar damage to the FRC network as in the F→M model, with a reduction of approximately 10-fold by day 21 compared with BMT recipients without GVHD. LEC numbers were also reduced at this time point but only by approximately 2-fold (Figure 3A). Clinical prevention of human GVHD can be achieved by selective removal of naive T cells from the graft, thus depleting T cells with the greatest potential for alloreactivity (34). Because CD62L expression is required for trafficking of naive T cells to LNs following experimental BMT (35), we reasoned that removal of CD62L^+^ cells from donor input T cells would also prevent damage to the FRC network. Transfer of CD62L^–^ T cells was effective at preventing GVHD in the B6→129 BMT model (Supplemental Figure 3A) and also induced significantly less depletion of the FRC population than nonmanipulated T cells (Figure 3B); this effect was also observed when the input T cell numbers were adjusted to allow equal representation of CD4^+^ and CD8^+^ T cell subsets between the experimental cohorts (data not shown). A second widely adopted strategy for preventing GVHD is the use of posttransplant cyclophosphamide (PTCy) which involves administration of a short pulse of cyclophosphamide, usually on days 3–4 following infusion of an unmanipulated graft (36). In preclinical models, PTCy depletes or inactivates rapidly dividing alloreactive T cells, while preserving nonalloreactive T cells and regulatory T cell (Treg) numbers (37, 38). We found that administration of cyclophosphamide at a dose of 25 mg/kg on days 3 and 4 following B6→129 BMT and T cell transfer partially reduced clinical GVHD scores and donor T cell expansion (Supplemental Figure 3B); however, the extent of FRC loss on day 18 was similar to GVHD controls (Figure 3C). We next addressed whether treatment of GVHD could allow subsequent recovery of the FRC population by adapting the B6→129 model to incorporate corticosteroids, as used routinely in the clinic (1). Thus, we treated BMT recipients with 0.3 mg/kg/day intraperitoneal dexamethasone or PBS starting from day 5 after BMT and cotransfer of donor T cells; FRC numbers were assessed 2 weeks after treatment initiation (day 19 after BMT). Dexamethasone treatment partially reduced the clinical GVHD score and donor T cell expansion, and led to a modest improvement in survival (Supplemental Figure 3C); however, FRC numbers fell to a similar extent as in GVHD control mice (Figure 3D). Of note, corticosteroid treatment alone over an equivalent period in non-BMT mice did not lead to reductions in FRC numbers, excluding any direct drug toxicity (Supplemental Figure 3D). Taken together with the results from Figure 2E, where robust CD8 depletion from day 5 offered almost full protection in the F→M BMT model, we reason that the failure of PTCy or corticosteroids to protect against FRC loss relate to their incomplete activity in blocking residual alloreactivity in the B6→129 model.

### Acute GVHD blocks stromal reorganization and repair of the FRC network.

**Figure 4:**
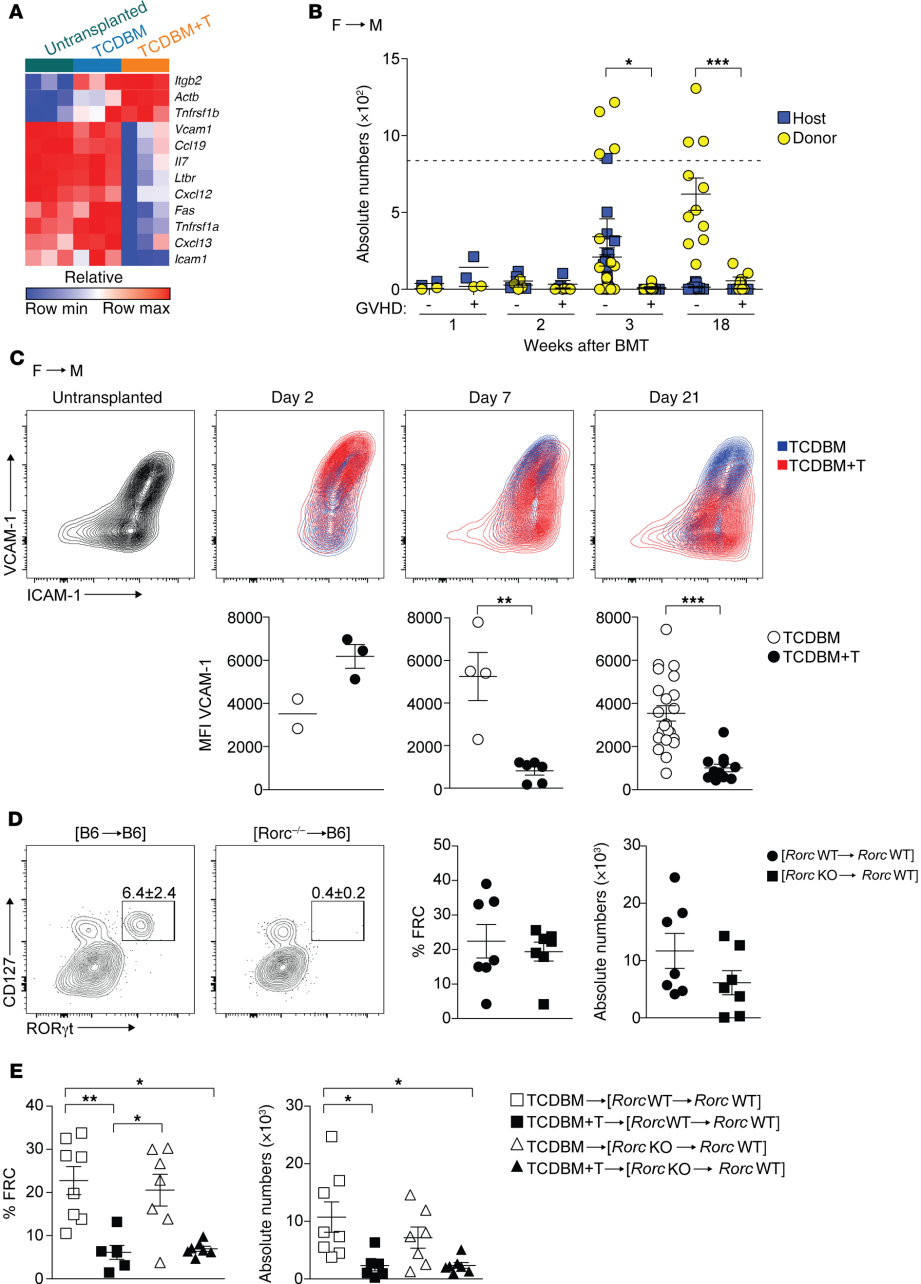
Acute GVHD blocks stromal reorganization and repair of the FRC network. (**A**) Heatmap depicting relative expression values of specific genes involved in a stromal reorganization program. Relative expression is shown in FRCs isolated from untransplanted mice, or from TCDBM recipients and TCDBM+T recipients 7 days following F→M BMT. (**B**) Absolute numbers of host and donor LTi cells were evaluated at indicated time points in the presence or absence of acute GVHD following F→M BMT. Dotted line indicates mean absolute numbers of LTi cells in untreated mice. Statistical analysis is of donor cells only. Data derived from 3 independent experiments. (**C**) Surface expression of VCAM1 and ICAM1 on CD45^–^CD31^–^gp38^+^ LN stromal cells in untransplanted controls (gray) and BMT recipients of TCDBM alone (blue) or TCDBM+T (red) at indicated time points after F→M BMT. Summary data depicting MFI of VCAM1 within the CD45^–^CD31^–^gp38^+^ population in TCDBM or TCDBM+T recipients is shown below the respective flow cytometry plots (data derived from 6 independent experiments). (**D**) [*Rorc* WT→*Rorc* WT] or [*Rorc* KO→*Rorc* WT] chimeras were analyzed for the presence of LTi cells within LNs at 8 weeks after primary BMT. Plots depict expression of CD127 and RORγt among lineage^–^CD117^+^ cells. Percentage and absolute numbers of FRCs is shown for [*Rorc* WT→*Rorc* WT] and [*Rorc* KO→*Rorc* WT] chimeras at 8 weeks after primary BMT (data derived from 3 independent experiments). (**E**) Eight weeks after the first BMT, [*Rorc* WT→*Rorc* WT] and [*Rorc* KO→*Rorc* WT] chimeras were reirradiated and underwent secondary F→M BMT with either TCDBM alone or TCDBM+T to induce acute GVHD. Plots show percentage (left) and absolute numbers (right) of FRCs analyzed at 2 weeks after the second transplantation (data derived from 2 independent experiments). Data represent mean ± SEM. *P < 0.05; **P < 0.01; ***P < 0.001 by Mann-Whitney *U* test (**B** and **C**) or Kruskal-Wallis ANOVA (**E**).

Our finding here that FRC population recovery was impaired following initial injury suggested the disruption of critical repair mechanisms required for LN stromal reconstruction. To determine whether FRC loss triggered a reorganizational transcriptional signature, as seen in acute viral infection (26), we flow sorted FRCs derived from mice developing acute GVHD and controls on day 7 in the Mh F→M model and performed RNA-seq. As shown in Figure 4A, the expression of reorganization genes (e.g., *Vcam1*, *Icam1*, *Cxcl13*, and *Ltbr*) was significantly reduced in GVHD mice compared with controls, suggesting that molecular interactions characteristic of crosstalk between LTo and LTi cells had been disrupted. To discern if GVHD-induced FRC injury provoked a similar influx of LTi cells as observed in viral infection, we next tracked numbers of LTi cells (defined as lineage^–^CD117^+^IL-7Rα^+^RORγt^+^) in both the F→M model and B6→129 model (see Supplemental Figure 4A for gating strategy), using congenic markers to identify their host/donor origin. LTi populations were negative for expression of NKp46 but positive for CCR6, consistent with their lymphoid organ location (Supplemental Figure 4A). As shown in Figure 4B (F→M model) and Supplemental Figure 4B (B6→129 model), the host LTi population was partially replaced over several weeks by donor-derived LTi cells in the absence of acute GVHD. In contrast, we observed a biphasic pattern in GVHD mice involving an initial trend for host LTi cells to be present in greater numbers (around day 7) compared with controls, but the almost complete elimination of the population (both host- and donor-derived) at later time points. To determine how acute GVHD would affect stromal reorganization, we evaluated BMT mice for the presence of activated LTo-like cells (defined as CD45^–^VCAM1^hi^ICAM1^hi^), akin to those required for embryonic LN development (19). As shown in Figure 4C (F→M model) and Supplemental Figure 4C (B6→129 model), acute GVHD led to an early trend (day 2 in the F→M model and day 7 in the B6→129 model) for an increase in the frequency of the CD45^–^VCAM1^hi^ICAM1^hi^ population but, in both models, this early increase was not sustained compared with BMT mice without acute GVHD (Figure 4C). One possible explanation for the failure to invoke a sustained FRC repair program in acute GVHD was the failure to maintain LTi cell numbers in the PLNs, thus impairing crosstalk with FRCs or their LTo-like precursors. To determine whether lack of host LTi cells would increase the extent of FRC loss, as reported for acute LCMV infection (26), we adapted our F→M model to eliminate host LTi cells by using recipient mice that lack RORγt (encoded by the gene *Rorc*), a transcription factor that is required for LTi cell development (39). Thus, we compared FRC numbers following induction of GVHD following secondary BMT in established [male *Rorc* WT→male *Rorc* WT] or [male *Rorc* KO→male *Rorc* WT] BM chimeras, the latter chimeras lacking LTi cells. As shown in Figure 4D, although host LTi cells were absent in established [*Rorc* KO→*Rorc* WT] chimeras before the second BMT, we observed no difference in FRC baseline numbers (Figure 4D). Furthermore, and in contrast to acute LCMV infection (26), the absence of host LTi cells did not increase the extent of FRC injury following subsequent induction of acute GVHD in a secondary F→M BMT (Figure 4E). Neither Mh T cell expansion nor acute GVHD severity was affected by the absence of RORγt^+^ cells (data not shown). Together, these data show that the normal repair mechanism for FRC restoration is profoundly impaired in acute GVHD. However, unlike acute LCMV infection, host LTi cells are redundant in protecting the FRC network from injury.

### Autoreactive T cells fail to be purged from the periphery in acute GVHD.

**Figure 5:**
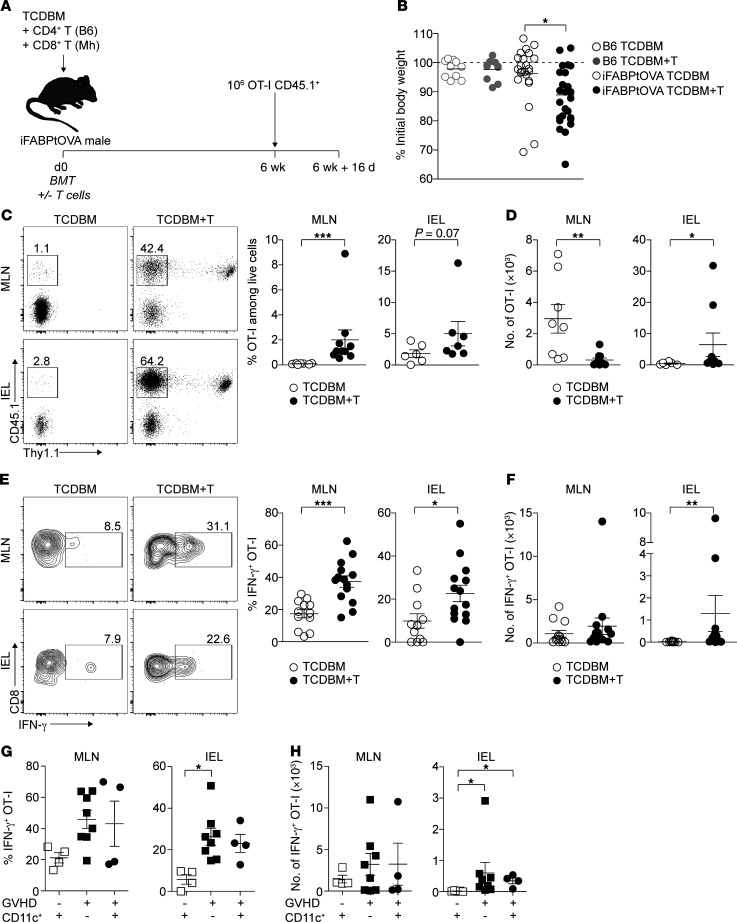
OVA-specific OT-I T cells fail to be purged from the periphery of iFABPtOVA mice with acute GVHD. (**A**) Murine transplantation model to investigate peripheral deletion of self-reactive T cells. Acute GVHD was induced in iFABPtOVA male BMT recipients by cotransfer of female TCDBM and Mh CD8^+^ T cells. No-GVHD controls received TCDBM alone. Male B6 recipients undergoing F→M BMT with or without acute GVHD served as further controls. Six weeks after BMT, 1 × 10^6^ OT-I T cells were transferred and mice were analyzed after 16 days (data derived from 8 independent experiments). (**B**) Weight change in recipient mice after OT-I T cell transfer is shown as percentage of initial body weight (defined as time point of OT-I transfer). (**C**) Flow cytometry plots depict surface expression of CD45.1 and Thy1.1 among CD8^+^ T cells (OT-I T cells are identified as CD45.1^+^Thy1.1^–^; Mh T cells are identified as CD45.1^+^Thy1.1^+^). Frequencies of OT-I T cells among total live cells in MLNs and the IEL are summarized in dot plots (right panel). (**D**) Absolute numbers of OT-I T cells in MLNs and the IEL (data derived from 7 independent experiments). (**E**) Flow cytometry plots depict intracellular IFN-γ expression among CD8^+^CD45.1^+^ OT-I T cells in MLNs and the IEL. Percentage of IFN-γ^+^ OT-I T cells is summarized in dot plots (right panel). (**F**) Absolute numbers of IFN-γ^+^ OT-I T cells in MLNs and the IEL (data derived from 8 independent experiments). (**G** and **H**) IFN-γ secretion was measured in acute GVHD^+^ recipients in the absence or presence of donor-derived CD11c^+^ DCs. Percentage (**G**) and absolute numbers (**H**) of IFN-γ^+^ OT-I T cells are shown for MLNs and the IEL (data derived from 3 independent experiments). Data represent mean ± SEM. *P < 0.05; **P < 0.01; ***P < 0.001 by Mann-Whitney *U* test (**C**–**F**) or Kruskal-Wallis ANOVA (**B**, **G**, and **H**).

Our findings that GVHD induced early PTA gene downregulation in FRCs (Figure 1, D and E) and subsequent elimination of almost the entire FRC population (Figures 2 and 3) suggested that intranodal display of PTAs would be severely disrupted. We reasoned that defects in PTA presentation would increase the risk that autoreactive T cells would develop effector functions capable of inducing tissue injury. To test this hypothesis, we used a model antigen system where PTA display by LN stromal cells is critical for peripheral tolerance of autoreactive T cells. Thus, we adapted the iFABPtOVA model in which a transgene encodes a truncated cytosolic form of OVA (tOVA) regulated by the promoter for intestinal fatty acid binding protein (iFABP) leading to the expression of the model self-antigen in intestinal epithelial cells (40). In this model, radioresistant LN stromal cells can directly present OVA to induce abortive proliferation and deletion of OVA-specific OT-I CD8^+^ T cells, thus preventing intestinal inflammation (15, 16). Among the LN stromal cells, expression of OVA is restricted to the FRC population, suggesting that this population is critical for tolerance (15). We hypothesized that development of acute GVHD in iFABPtOVA mice would abrogate this putative tolerance mechanism as a result of FRC depletion and loss of LN display of the model PTA. We therefore induced acute GVHD in iFABPtOVA male BMT recipients by cotransfer of female TCDBM and Mh CD8^+^ T cells (Figure 5A); additional male OVA-negative B6 recipients undergoing F→M BMT with or without acute GVHD served as controls. Similarly to nontransgenic BMT recipients, development of acute GVHD in iFABPtOVA mice reduced total FRC numbers by 14 days after BMT compared with GVHD^–^ iFABPtOVA controls (Supplemental Figure 5A); intranodal expression of the model self-antigen OVA by residual FRCs was also reduced in GVHD mice (Supplemental Figure 5A). At 6 weeks following BMT, when the acute effects of irradiation had resolved, the integrity of the mechanism underlying peripheral tolerance to intestinal OVA was evaluated in each group by transferring 1 × 10^6^ OT-I T cells, which were then tracked as a surrogate for autoreactive T cells (Figure 5A). By day 16 following OT-I T cell transfer, GVHD^+^ iFABPtOVA BMT recipients showed significant weight loss compared with GVHD^–^ iFABPtOVA controls (Figure 5B). OT-I transfer had no effect on the weight of OVA-negative GVHD^+^ B6 recipients, indicating that the weight loss in GVHD^+^ iFABPtOVA mice was antigen specific (Figure 5B). To investigate whether weight loss in GVHD^+^ iFABPtOVA BMT recipients was due to a failure to purge transferred OT-I effector cells from the periphery, we measured OT-I numbers and functions in LNs and small intestine. As shown in Figure 5C, transferred OT-I T cells were detectable at significantly higher frequencies in the PLNs (Supplemental Figure 5B), mesenteric LNs (MLNs), and the intraepithelial lymphocyte compartment (IEL) of GVHD^+^ iFABPtOVA mice compared with GVHD^–^ iFABPtOVA controls (Figure 5C; B6 BMT controls with and without GVHD are shown in Supplemental Figure 5C). Consistent with the effect of the disruption of the FRC network on LN integrity and the expected reduction in global LN T cell numbers (24, 25), absolute numbers of OT-I T cells were lower in the MLN GVHD^+^ iFABPtOVA mice than in GVHD^–^ controls; however, OT-I absolute numbers were significantly increased in the small intestine IEL (Figure 5D). To determine the functions of the OT-I T cell population, we measured cytokine generation by OT-I T cells in PLNs (Supplemental Figure 5B), MLNs, and the IEL from each group following brief ex vivo re-stimulation. As shown in Figure 5, E and F, MLN and IEL OT-I T cells from GVHD^+^ iFABPtOVA mice expressed higher quantities of IFN-γ compared with controls, indicating a failure to block T cell autoreactivity in acute GVHD. OT-I frequency and absolute numbers were not increased in the small intestine IEL of OVA-negative GVHD^+^ B6 BMT recipients, indicating that bystander expansion and trafficking of OT-I cells in the absence of antigen did not occur (Supplemental Figure 5C).

Although tolerance in the iFABP-tOVA model occurs through peripheral deletion, surviving OT-I T cells may still retain the ability to induce intestinal injury if cross-primed by professional antigen-presenting cells during an unrelated inflammatory process (40, 41). Furthermore, it has recently been shown that donor-derived, migratory CD103^+^CD11b^–^ dendritic cells (DCs) can aggravate intestinal inflammation in acute GVHD by cross-presenting host antigens in the early phase (<2 weeks) following allogeneic BMT (42). We therefore sought to determine whether cross-presentation of intestinal OVA antigen by donor DCs could also be disrupting loss of peripheral tolerance in GVHD^+^ iFABPtOVA mice by adapting the experimental model to allow depletion of donor DCs. Thus, irradiated iFABPtOVA mice were reconstituted with BM from CD11cDTR mice (43), allowing the specific depletion of CD11c^+^ DCs upon DT administration at the point of transfer of OT-I T cells 6 weeks following BMT. As shown in Figure 5, G and H, depletion of DCs by intraperitoneal injection of DT every third day starting on day 41 following BMT (Supplemental Figure 5D) and transfer of OT-I T cells on day 42 had no effect on the subsequent effector function of OT-I measured by secretion of IFN-γ in MLNs and the IEL 16 days later. Thus, both in the absence and presence of donor DCs, OT-I T cells isolated from GVHD^+^ iFABPtOVA recipients showed equivalent elevation in IFN-γ expression compared with GVHD^–^ iFABPtOVA mice. Taken together, these data demonstrate a failure to purge autoaggressive T cells in the periphery following the development of acute GVHD through mechanisms that are independent of enhanced cross-presentation of self-antigens by donor DCs.

### Loss of FRCs is sufficient to break peripheral tolerance of autoreactive T cells.

**Figure 6:**
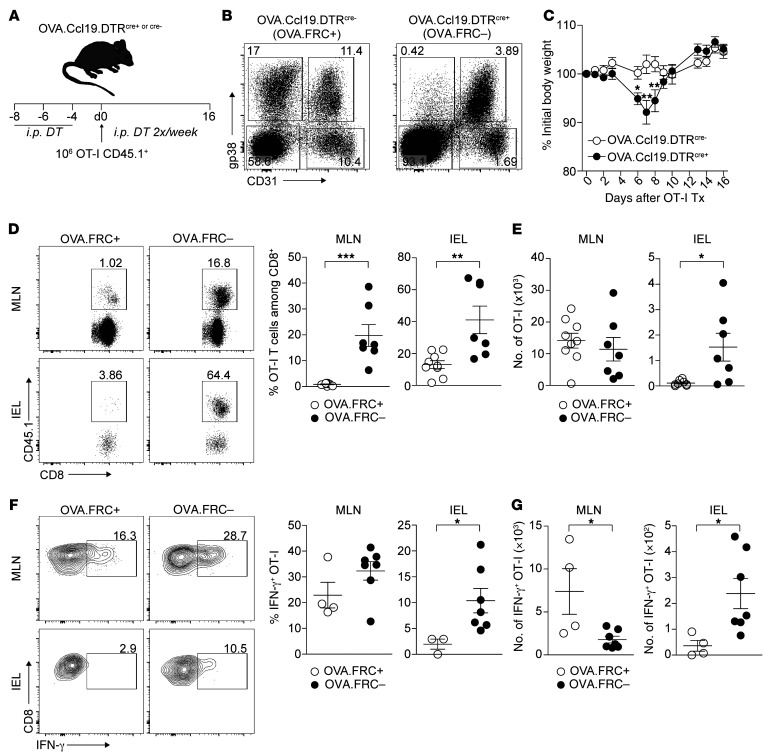
Specific depletion of FRCs is sufficient to break peripheral tolerance of autoreactive T cells in steady state. (**A**) OVA.Ccl19.DTRcre^+^ and cre^–^ mice received 500 ng DT i.p. on days –8, –6, and –4. OT-I T cells (1 × 10^6^) were transferred on day 0 and mice were analyzed on day 16. (**B**) FRC depletion in OVA.Ccl19.DTRcre^+^ versus cre^–^ mice upon DT treatment. Flow cytometry plots depict surface expression of gp38 and CD31 among CD45^–^ LN stromal cells. (**C**) Weight change in OVA.Ccl19.DTRcre^+^ and cre^–^ mice is shown as percentage of initial body weight (defined as time point of OT-I transfer; data derived from 4 independent experiments). (**D**) OT-I T cells were identified as CD8^+^CD45.1^+^. Percentages of OT-I T cells are shown for MLNs and the IEL and summarized in dot plots (right). (**E**) Absolute numbers of OT-I T cells in MLNs and the IEL (data derived from 4 independent experiments). (**F**) IFN-γ secretion measured by intracellular flow cytometry. IFN-γ expression is shown among CD8^+^CD45.1^+^ OT-I T cells. Percentage of IFN-γ^+^ OT-I T cells in MLNs and the IEL is summarized in dot plots (right). (**G**) Absolute numbers of IFN-γ^+^ OT-I T cells in MLNs and the IEL (data derived from 3 independent experiments). Data represent mean ± SEM. *P < 0.05; **P < 0.01; ***P < 0.001 by Mann-Whitney *U* test.

Because multiple mechanisms could potentially explain loss of peripheral tolerance in the context of inflammation, it was possible that FRC network injury was unrelated to the autoaggressive behavior of OT-I T cells in GVHD^+^ iFABPtOVA mice. We therefore asked whether the loss of FRCs in a noninflammatory environment in mice without GVHD would be sufficient to disrupt peripheral tolerance induction in LNs. We therefore crossed iFABPtOVA and Ccl19cre.DTR (24) (OVA.Ccl19.DTR) mice to allow for DT-mediated depletion of *Ccl19^+^* FRCs in the absence of GVHD. During the 3-week course of the experiment, DT treatment of OVA.Ccl19.DTR mice on days –8, –6, –4, +2, +6, +9, and +13 led to long-lasting (>95%) depletion of FRCs (Figure 6, A and B). As shown in Figure 6C, transfer of OT-I cells to recipient mice led to transient weight loss in the FRC-depleted cohort, peaking at day 7. As we observed in GVHD^+^ iFABPtOVA recipients, transferred OT-I T cells were detectable at significantly higher frequencies in both MLNs and the IEL of FRC-depleted hosts compared with FRC-replete controls (Figure 6D). Similar to the GVHD model, absolute numbers of OT-I T cells were also increased in the IEL when FRCs were depleted (Figure 6E) and this was associated with increased numbers of IFN-γ–secreting cells in both the MLNs and IEL (Figure 6, F and G). Thus, these experiments identify the FRC population as being specifically required for peripheral tolerance of autoreactive CD8^+^ T cells in the iFABPtOVA model. Furthermore, these data show that FRC elimination is sufficient to trigger autoaggressive T cell behavior even in the absence of inflammation, a scenario where other peripheral tolerance mechanisms are anticipated to remain intact. Thus, although GVHD may disrupt multiple regulatory mechanisms in the periphery, these data indicate that FRC network damage contributes to the loss of tolerance to PTAs.

### FRCs express a distinct PTA gene signature enriched for genes normally expressed in target organs of chronic GVHD.

**Figure 7:**
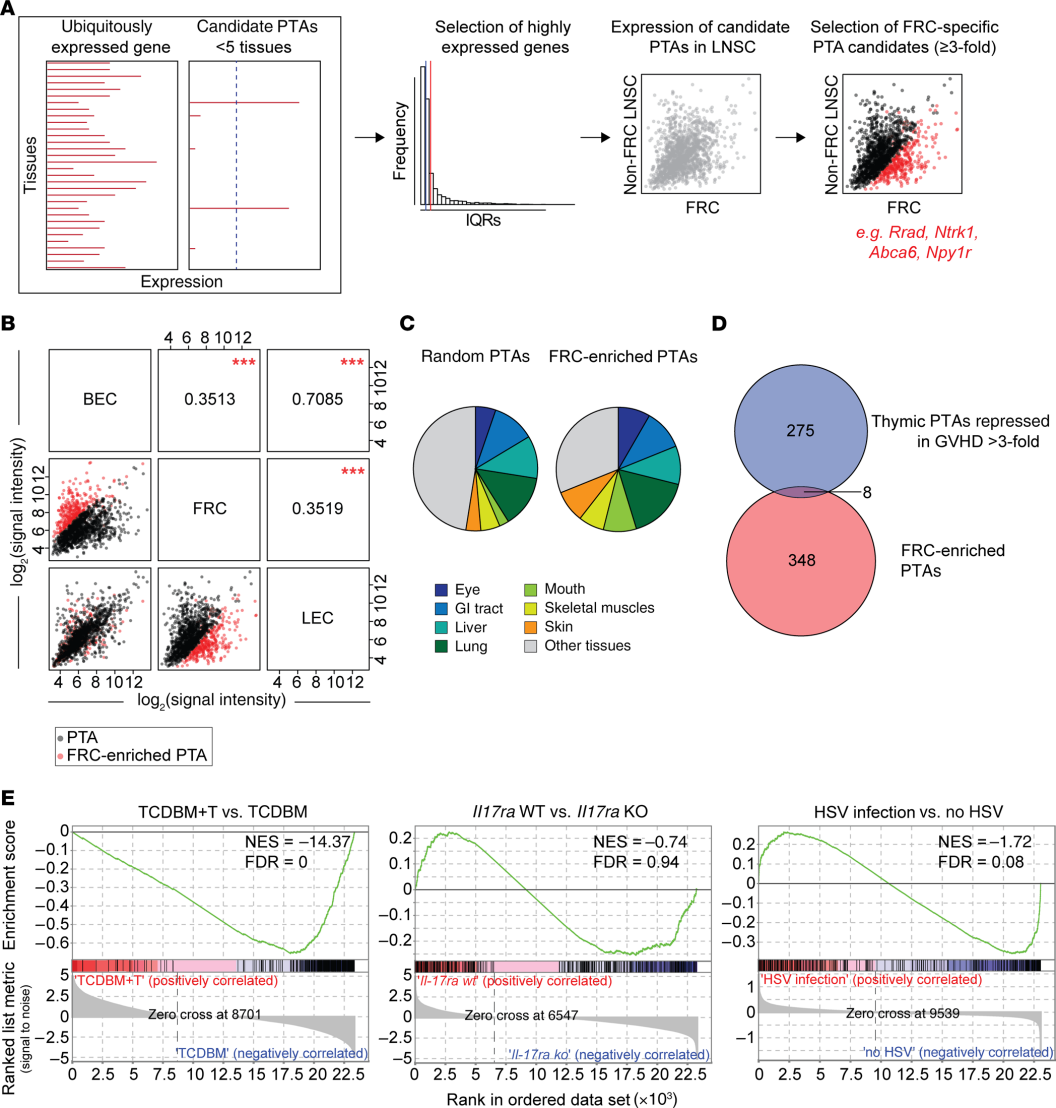
FRCs express a distinct PTA gene signature enriched for genes normally expressed in target organs of chronic GVHD. (**A**) Analytical pipeline of FRC-specific PTA candidate.. LNSC, lymph node stromal cell. (**B**) Correlation matrix of PTA candidates in published gene expression data from different LN stromal cell subsets. Pearson’s correlation coefficient *r* between the respective subsets is indicated. FRC-enriched PTA genes are highlighted in red. ***P < 0.001, indicating the quality of each measured Pearson’s *r* between any given subset. (**C**) Tissue representation of FRC-enriched PTAs (≥3-fold expression compared with other LN stromal cell subsets). As control, a random set of 356 PTAs, which are not enriched in FRCs, was used. (**D**) Venn diagram showing the overlap between thymic PTAs repressed in GVHD by greater than 3-fold and PTAs identified to be enriched in FRCs compared with other LN stromal subsets. (**E**) Enrichment of the FRC-enriched PTA gene set was analyzed by GSEA in FRCs isolated from GVHD^+^ versus GVHD^–^ recipients, or from vaccinated mice in which FRCs selectively lacked IL-17Rα versus wild-type controls, or from HSV-infected versus control noninfected mice.

Because it has been shown that individual LN stromal cell subsets each express a distinct repertoire of PTAs (14, 15), we next sought to characterize the nature of an FRC-specific PTA signature in nontransgenic mice by analyzing transcriptional profiles for individual, steady-state LN stromal cell subsets using published data from the Immgen consortium (29) (a summary of the analytical pipeline is shown in Figure 7A). Putative PTAs were identified on the basis of their transcription in fewer than 5 different tissues (9, 12, 44) and gene expression within the top quartile for pooled LN stromal cell data (*n* = 1494 genes). PTA gene expression in FRCs was distinct from LECs and BECs, whereas LEC and BEC PTA gene expression was very similar (Figure 7B). Based on a comparison of PTA gene expression between the different LN stromal cell subsets (Figure 7B), we identified a list of 356 putative FRC-specific PTA genes based on greater than 3-fold higher expression and an adjusted *P* value of 0.05 or less compared with the other LN stromal subsets (listed in Supplemental Table 1). Of note, when we evaluated in which tissues each of these genes was expressed, we found that 246 of 356 (69.1%) of the PTA genes were also expressed in known target tissues affected by chronic GVHD (skin epidermis, cornea, lacrimal gland, gut, liver, salivary gland, tongue epidermis, skin epidermis, lung, and skeletal muscle); this same level of enrichment was not observed in a randomly selected set of 356 non–FRC-specific PTA genes expressed in LN stromal cells where 187 of 356 (52.2%) genes were expressed in chronic GVHD target tissues (*P* < 0.0001, Fisher’s exact test; Figure 7C). We also compared the FRC PTA gene set with 283 PTA genes that are normally expressed by thymic mTECs but whose expression is reduced (>3-fold) following the onset of acute GVHD (9). Although the overlap in gene sets was slightly greater than expected — 8 overlapping genes from a total of 6611 PTA genes (44), a 2.1-fold increase over expected (*P* = 0.012 by hypergeometric testing) — the vast majority of genes (>97%) were specific to either the mTEC or FRC compartment (Figure 7D). Akin to the acute downregulation of known PTA genes shown in Figure 1E, GSEA showed acute downregulation of the FRC-specific PTA gene set in FRCs sorted on day 7 following the onset of acute GVHD in the F→M BMT model (NES –14.4, FDR *q* value = 0; Figure 7E). On day 7, the majority of the FRC population remained intact (Figure 2B) and these early reductions in gene expression were not indicative of a global depression in gene transcription because expression of a random list of non-PTA genes in FRCs showed heterogeneous changes in gene expression compared with controls (Supplemental Figure 6). Furthermore, PTA gene expression was also specific to acute GVHD and did not occur when FRCs were exposed to other inflammatory stimuli (Figure 7E). Taken together, these data indicate that acute GVHD rapidly disrupts intranodal display of a unique PTA gene set that mirrors the repertoire of genes expressed in the target organs of chronic GVHD.

## Discussion

We have shown that acute GVHD damages the FRC network in LNs, as well as its capacity for regeneration. Unlike FRC loss accompanying acute LCMV infection, which is associated with a rapid influx of LTi cells and induction of LN reorganization (26), no similar process is evident in GVHD and LTi cells provide no protection. While FRC loss is known to be associated with immune deficiency (24, 25), we find here that the same process can also lead to simultaneous autoimmunity. Thus, early downregulation of putative PTA gene expression in FRCs, followed later by physical loss of the FRC network, impedes peripheral education of autoreactive T cells and allows tissue injury to occur. Together with the known impact of acute GVHD on thymic education, our data provide support for a 2-hit model in which defects in tissue-restricted antigen display in both the thymus and lymph node allow autoimmunity to break through and perpetuate chronic inflammation.

FRC network injury was consistently observed in several models of acute GVHD, although in contrast to a previous report (21) we also observed FRC damage in a purely CD4^+^ T cell–dependent model, consistent with the upregulated expression of MHC class II on FRCs in the context of an inflammatory stimulus (29). These data indicate an inherent FRC sensitivity to immune injury compared with other LN stromal cell populations. Although there were some differences, we observed very similar changes in gene expression in FRCs early following acute HSV infection (27), including increased representation of pathways related to apoptosis. In contrast to GVHD, HSV infection is not ultimately associated with net loss of the FRC network. Thus, our findings suggest default components of the transcriptional response of FRCs to diverse inflammatory stimuli; these changes are consistent with a requirement for FRC population expansion to accommodate a rapidly developing immune response and a counterregulatory increase in susceptibility to apoptosis allowing a subsequent return to homeostasis. However, in the context of acute GVHD, homeostasis cannot be restored because of ongoing immune injury and/or the failure of physiological FRC network repair mechanisms.

It has been proposed that FRC network repair following acute LCMV infection triggers a reorganizational program that recapitulates the LTi-LTo interaction required for LN organogenesis in the embryo (26). We explored whether a similar process occurs following injury imposed by acute GVHD. Although host LTi cells were radiosensitive, we observed an initial trend for greater LTi cell persistence compared with controls as well as a transient increase in the frequency of a CD45^–^VCAM^hi^ICAM^hi^ population akin to the phenotype of activated LTo-like cells; however, neither population was sustained and both cell types were severely depleted compared with no-GVHD controls at later time points. Our data therefore indicate that FRC injury in acute GVHD leads to an abortive reorganizational program distinct from the process occurring following acute LCMV infection. Indeed, we found that host-derived LTi cells were redundant in protecting the FRC population, unlike the situation reported for acute LCMV (26). Similarly, we observed no increase in the frequency of LTi or VCAM1^hi^ICAM1^hi^ cells following transient depletion of FRCs in Ccl19.DTR mice where network destruction occurs in the absence of inflammation (data not shown). There is, therefore, a need to better define the constituent elements that normally permit stromal regeneration in adults according to the precise context in which FRC network degeneration occurs.

Although the global transcriptional response of FRCs in acute GVHD and other inflammatory stimuli was similar overall, acute downregulation of a putative FRC-specific PTA gene set occurred only following the onset of acute GVHD. Protein expression of PTAs in LN stromal populations is usually below the limits of detection so that the peripheral display of PTAs other than the model antigen we used has to be inferred. The ectopic expression of genes encoding PTAs in pancreatic LN stromal cells is regulated in part by DEAF1 (18); this transcriptional regulator has structural homology to AIRE, containing a DNA-binding SAND (Sp100, AIRE-1, NucP41/75, and DEAF1) domain, which mediates chromatin-dependent transcription and protein-protein interactions and a ZF-MYND (zinc finger, myeloid, Nervy, and DEAF1) domain that is similar to the plant homeodomain 1 of AIRE. DEAF1 controls the transcription of PTA but also regulates their processing and presentation by controlling expression of eukaryotic translation initiation factor 4 gamma 2 (45). *Deaf1* gene expression was reduced early in FRCs following acute GVHD onset and this change was coincidental with reduced PTA gene expression. Indeed, we found a significant overlap between genes downregulated in LN stroma of *Deaf1*-knockout mice and FRCs in acute GVHD, although this comparison is limited by differences in mouse strains used to identify regulated genes (BALB/c for *Deaf1* knockout because B6 mice are not viable after birth) and the relative lack of purity of the stroma analyzed (18). The lack of overlap between the FRC-specific set of 356 PTA genes we defined here and the gene set previously identified as being regulated by DEAF1 (data not shown) may also relate to these same issues, although other mechanisms may exist for PTA gene regulation in the LNs. Future studies involving FRC-specific deletion of *Deaf1* will provide further insight as to whether its downregulation in acute GVHD is solely responsible for reduced PTA expression. Furthermore, the recent finding that LN innate lymphoid cells type 3 (ILC3) express AIRE (46) and the permanent depletion of the ILC3-related LTi population in GVHD shown here could suggest a more general deficit in peripheral antigen display than accountable by injury to the FRC network alone.

The FRC PTA gene set we defined from steady-state FRCs was distinct from that expressed by other LN stromal populations and enriched for genes expressed in the classical target organs of chronic GVHD. Because there are no available GVHD models that fully recapitulate the transition from acute to chronic GVHD, we employed the more tractable iFABPtOVA system to test the hypothesis that loss of FRC antigen display would increase the frequency and functions of autoreactive T cells (40). This model system was chosen because radioresistant LN stromal cells have been shown to directly present OVA to induce tolerance through abortive proliferation and deletion of OVA-specific OT-I CD8^+^ T cells (15, 16). We showed that purging of autoreactive CD8^+^ T cells from the peripheral repertoire in iFABPtOVA mice was abrogated several weeks following the onset of acute GVHD through a mechanism that was independent of increased cross-presentation of OVA by DCs in gut-draining LNs. Although it is likely that the inflammatory environment of GVHD impairs other peripheral regulatory mechanisms (e.g., Tregs), depletion of FRCs in the absence of inflammation was able to reproduce the same degree of autoreactivity as observed in GVHD, indicating that this mechanism alone is sufficient to break tolerance. It is important to note that we evaluated this loss of peripheral tolerance in only 1 model system where alloreactive T cells (HY-specific Mh), autoreactive T cells (OT-I), and recipient mice expressing a model PTA in FRCs (iFABPtOVA) were all on a B6 background. It will be important, therefore, to validate our findings in an independent model involving other PTAs or other strain backgrounds; in turn, this will depend on the development of tractable systems where autoreactive T cells with reactivity to FRC-expressed PTAs can be tracked. How GVHD-induced disruption to the LN architecture affects other regulatory mechanisms will also be important to explore, particularly in relation to stromal MHC class II–restricted expression of PTAs or the survival of other regulatory populations. For example, it has recently been reported that FRCs can suppress intestinal injury by regulating the functions of ILC1 in gut-associated lymphoid tissue when triggered by inflammation (47). Although this mechanism could conceivably contribute to evolution of intestinal GVHD, ILC1 also require steady state transpresentation of IL-15 by FRCs for their survival (47); thus, the loss of tolerance in iFABPtOVA mice we observed following inducible FRC depletion in the absence of an inflammatory stimulus (Figure 6) is unlikely to be dependent on ILC1 activation.

A key question is how current or emerging clinical strategies to prevent or treat GVHD will affect the FRC population and peripheral tolerance. Individual strategies will differ in the extent to which they prevent initial damage to the FRC network, block ongoing immune injury, or preserve repair/regeneration pathways. It is unlikely, therefore, that this process is all-or-none and we reason that the level of injury caused by GVHD will vary in human patients according to multiple factors. In addition, the animal models presented here do not fully recapitulate the clinical setting (e.g., the use of calcineurin inhibitor drugs) or were found to be associated with incomplete inhibition of the alloreactive response (e.g., as shown for PTCy and corticosteroid treatment). Furthermore, some approaches may be better at promoting other peripheral tolerance mechanisms (e.g., expansion of Tregs with PTCy; refs. 37, 38) that could compensate for loss of peripheral education of autoreactive T cells by FRCs. Indeed, it is also possible that such tolerance mechanisms will ultimately enable regenerative/repair pathways to reemerge and restore FRC integrity even if initial injury has occurred.

In conclusion, our data highlight the importance of loss of regeneration/repair mechanisms as being an important driver of GVHD. While injury to epithelial stem cells in acute GVHD is the most overt example of this phenomenon (48, 49), lack of stromal repair within lymphoid organs (both thymus and LNs) disrupts the normal mechanisms of T cell development and education leading to the paradoxical coexistence of immunodeficiency and autoimmunity. Future strategies designed to ensure continuous PTA display in the periphery by protection or regeneration of LN stroma may be essential to breaking the transition from acute to chronic GVHD.

## Methods

### Mice.

C57BL/6 (B6), 129/Sv, and *Pfp^–/–^* mice were purchased from Charles River Laboratories and bred in house by UCL Biological Services. *B6 CD45.1* mice, OT-I *Rag1^–/–^* mice, *CD11c.DTR* mice, *B2m^–/–^* mice, and ROSA26.iDTR mice were purchased from the Jackson Laboratory. Marilyn (50) and MataHari mice (51) were provided by Jian Chai (Imperial College London, London, United Kingdom [UK]) and bred in house. iFABPtOVA mice were originally generated by Leo Lefrançois at the University of Connecticut (40) and provided by Simon Milling (University of Glasgow, UK). *Ccl19.cre* mice were provided by Burkhard Ludewig (Kantonal Hospital St. Gallen, Switzerland) (24) and bred in house by crossing with iFABPtOVA and/or ROSA26.iDTR mice.

### Flow cytometry.

The following anti-mouse surface antibodies (with clone numbers) were used: CD45 (30-F11, BioLegend), gp38 (8.1.1, BioLegend), CD31 (MEC13.3, BioLegend), ICAM1 (YN1/1.7.4, BioLegend), VCAM1, (429 [MVCAM.A], BioLegend), CD8α (53-6.7, BD Biosciences), CD4 (RM4-4, eBioscience), Vα2 (B20.1, eBioscience), Vβ5 (MR9-4, eBioscience), CD45.1 (A20, BD Biosciences), CD90.1 (Thy1.1, HIS51, eBioscience), CD62L (MEL-14, BD Biosciences), CD44 (IM7, BioLegend), CD69 (H1.2F3, BD Biosciences), Vβ8.3 (1B3.3, BD Biosciences), CD11c (HL3, BD Biosciences), CD11b (M1/70, BioLegend), IA-IE (MHCII, M5/114.15.2, eBioscience), CD24 (M1/69, BD Biosciences), CD127 (A7R34, eBioscience), CD117 (2B8MH, BD Biosciences), CD3 (145-2C11, BD Biosciences), CD19 (1D3, BD Biosciences), NK1.1 (PK136, eBioscience), LY6G (1A8, BD Biosciences) CD196 (CCR6; 29-2L17, BioLegend), NKp46 (29A1.4, eBioscience) and F4/80 (BM8, eBioscience). For intracellular cytokine staining, cells were incubated with 10 ng/mL phorbol 12-myristate 13-acetate (PMA) (Sigma-Aldrich) and 1 μg/mL ionomycin (Sigma-Aldrich) in the presence of 1 μg/ml brefeldin A (Sigma-Aldrich) for 4 hours at 37°C before fixation and permeabilization (Cytofix/Cytoperm, BD Biosciences), followed by incubation with antibody against IFN-γ (XMG1.2, BD Biosciences). Intracellular RORγt (Q31-378, BD Biosciences) staining was carried out in nonstimulated cells using the Foxp3/Transcription Factor Staining Buffer Set (eBioscience). Cells were acquired on a BD LSRFortessa cell analyzer and analyzed using FlowJo v10 (Tree Star). Cells were sorted on a BD FACSAria IIu cell sorter.

### BMT.

Single miHA mismatch HY-specific model: BMT was performed as described previously (30, 32). Briefly, to induce acute GVHD, male C57BL/6 (B6; H-2^b^) or iFABPtOVA (H-2^b^) recipient mice were lethally irradiated (11 Gy total body irradiation) and reconstituted with 5 × 10^6^ TCDBM cells, 2 × 10^6^ polyclonal female B6 CD4^+^ T cells, and 1 × 10^6^ female Mh CD8^+^ T cells. No-GVHD control recipients received TCDBM alone. Multiple miHA mismatch model: 129/Sv (129; H-2^b^) mice were used as recipients. Acute GVHD was induced by transferring 5 × 10^6^ TCDBM cells, 2 × 10^6^ CD4^+^, and 1 × 10^6^ CD8^+^ T cells (all from B6 donors). No-acute-GVHD controls received TCDBM alone. T cell depletion of BM through negative selection and isolation of CD4^+^ and CD8^+^ T cells (including OT-I cells) by positive selection was performed using manual MACS Cell Separation (CD4 [L3T4] MicroBeads and CD8α [Ly-2] MicroBeads; Miltenyi Biotec) according to the manufacturer’s instructions. Negative selection of CD62L^+^ cells was performed using CD62L microbeads (Miltenyi Biotec). For in vivo CD8 depletion, 1.44 mg of anti-CD8α depleting antibody (Bio X Cell) was given by i.p. injection and repeated after 2 weeks. Dexamethasone (Wockhardt) treatment started on day 5 after transplant and was given daily by i.p. injections (0.3 mg/kg/day) until takedown. For PTCy experiments, cyclophosphamide (Sigma-Aldrich) was given by i.p. injection on days 3 and 4 at 25 mg/kg/day.

### Cell isolation.

LN stromal cells were isolated from PLNs and MLNs at indicated time points after transplantation or DT treatment and enzymatically digested with 0.2 mg/mL Liberase (Roche Diagnostics) and 20 μg/mL DNase (Sigma-Aldrich) in PBS at 37°C for 30 minutes with mechanical disruption every 5 minutes. Supernatants containing stroma cells were collected and the enzyme mix was replaced. LN, spleen, and small intestine cells were isolated at indicated time points using methods described previously (30, 32). Cells were counted using a CASY Model TT Cell Counter and Analyzer (Roche).

### RT-PCR.

RNA was isolated from sorted FRCs using the RNeasy MicroKit (Qiagen) according to the manufacturer’s protocol. RNA was transcribed into cDNA using QuantiTect Reverse Transcription (Qiagen). Quantitative real-time PCR was performed on a CFX96 Touch Real-Time PCR Detection System (Bio-Rad) using QuantiFast SYBR Green (Qiagen). The following primers were used: *Il7*, (for) 5′-GTTCCATGGTACTAGCGAACCAA-3′, (rev) 5′-GGATGCGGTGTCTCTAGCTG-3′; Ccl19, (for) 5′-ACTTGCACTTGGCTCCTGAAC-3′, (rev) 5′-GTGAGCCTGAGAGACTGTGTG-3′; *Deaf1*, (for) 5′-ACTCTGAGTGGCCCTGTCAG-3′, (rev) 5′-TGTCAAAGGTCAGTGCTCC-3′; *Mlana*, (for) 5′-CTGCTGAAGAGGCCGCAGGG-3′, (rev) 5′-GGAGCGTTGGGAACCACGGG-3′; *Rrad*, (for) 5′-GGGAACAGGATGGGGGCTGC-3′, (rev) 5′-TGGCGCGGAAGGCCATCTTG-3′; *Plp*, (for) 5′-CAGGGGGCCAGAAGGGGAGG-3′, (rev) 5′-GCAGCACCCACAAACGCAGC-3′; *Ova*, (for) 5′-CACAAGCAATGCCTTTCAGA-3′, (rev) 5′-GAATGGATGGTCAGCCCTAA-3′; *Gapdh*, (for) 5′-GGTGAAGGTCGGTGTGAACG-3′, (rev) 5′-ACCATGTAGTTCAGGTCAATGAAGG-3′.

### RNA-seq and analysis.

RNA was amplified using the SMART Seq v4 Ultra Low Input RNA kit (Takara Bio) and cDNA libraries were prepared according to the Nextera XT DNA Library Preparation kit protocol (Illumina). Sequencing was performed on an Illumina NextSeq 500, generating more than 15 million 38-bp paired-end reads per sample. Adapter trimming of the reads was performed by the FASTQ Toolkit. Alignment and library mapping were performed using TopHat Alignment and Cufflinks Assembly & DE. Gene expression levels and differentially expressed genes were calculated using Cufflinks/Cuffdiff. Data are available on www.ebi.ac.uk/arrayexpress, accession number E-MTAB-8255.

### Identification of PTAs.

PTA genes were defined as genes being expressed in fewer than 5 tissues, as previously described (9, 44). Briefly, gene expression data from the public database Mouse GNF Mouse GeneAtlas v3 (http://biogps.org) were used to define PTAs. The published list of PTAs with tissue-restricted expression by Sansom et al. was used for downstream analysis (44). To analyze PTA expression in LN stromal cells, published microarray data were used (29). PTAs with high expression in FRCs (top quartile) and greater than 3-fold higher expression compared with other LN stromal cell subsets were defined as “FRC-enriched.”

### GSEA.

One hundred fifty-seven genes were identified as DEAF1 dependent based on greater than 3-fold higher expression in wild-type animals compared with *Deaf1*-KO animals and defined as the “DEAF1 gene set” (18). Eighty-four out of 157 genes were present in RNA-seq data from FRCs isolated from mice with or without acute GVHD. Enrichment of the DEAF1 gene set in TCDBM vs. TCDBM+T FRCs was performed using GSEA software (52). GSEA of naive FRCs, FRCs after HSV-1 infection or IL-17 exposure, and FRCs isolated from GVHD^+^ or GVHD^–^ recipients was performed with the gene sets derived from the REACTOME database collected in the Molecular Signatures Database (MSigDB v5.1). Network visualization was performed using Cytoscape (53).

### FRC and DC ablation in vivo.

iFABPtOVA, ROSA26.iDTR, and Ccl19.cre mice were crossed to generate OVA.Ccl19.DTR mice. Cre^+^ and Cre^–^ control mice were injected i.p. with 500 ng of DT on day –8, –6, and –4 prior to OT-I T cell transfer. To maintain FRC ablation, recipients received 500 ng DT i.p. on day 2, 6, 9, and 13. Mice were analyzed on day 16. For depletion of donor CD11c, BMT recipient mice received 5 × 10^6^ BM cells from CD11c.DTR donor mice together with polyclonal CD4^+^ and Mh CD8^+^ T cells. Six weeks after BMT and day –1 before OT-I T cell transfer, donor DCs were depleted upon i.p. injection of 100 ng DT (54). To maintain DC ablation, recipients received 100 ng DT i.p. every 72 hours.

### Immunofluorescence and confocal imaging.

For analysis of LN stromal cells by confocal microscopy, LNs were isolated on day 7 after transplantation and frozen in OCT (Cellpath). Sections (8 μm) were cut on a cryostat, dried, and fixed with acetone (–20°C). Primary antibodies were CD31-FITC (clone MEC13.3, BD Biosciences) and gp38-biotin (eBio8.1.1, eBioscience). Secondary antibodies were anti-FITC Alexa 488 (Life Technologies) and Streptavidin eFluor 570 (eBioscience). Sections were stained with DAPI and mounted with ProLong Diamond Antifade Mountant (Life Technologies). All images were captured on a Nikon Ti inverted microscope using a C2 confocal scan head (Nikon Instruments). Images were acquired with a 40× (Plan Apochromat N.A. 0.095 W.D. 0.21 mm) objective. Image analysis was done using the software ImageJ (NIH).

### Statistics.

The nonparametric unpaired Mann-Whitney *U* test was used for 2-group comparisons, whereas for multiple group comparisons Kruskal-Wallis 1-way ANOVA with Dunn’s post hoc test was performed using GraphPad Prism. A 2-tailed *P* value less than 0.05 was considered significant.

### Study approval.

All procedures were conducted in accordance with the UK Home Office Animals (Scientific Procedure) Act of 1986, and were approved by the Ethics and Welfare Committee of the Comparative Biology Unit, Hampstead Campus, University College London, London, UK.

## Author contributions

SD conceived the ideas, designed and performed experiments, analyzed and interpreted data, and wrote and reviewed the manuscript. PE conceived the ideas, designed and performed experiments, and analyzed and interpreted data. PSS, TM, and IRF performed experiments and analyzed data. HJS and CLB provided technical and material support and reviewed the manuscript. RC conceived the ideas, supervised the study, developed methodology, and wrote the manuscript.

## Supplementary Material

Supplemental data

## Version 1 01/09/2020

In-Press Preview

## Version 2 03/09/2020

Electronic publication

## Version 3 04/01/2020

Print issue publication
