# Supplementary material for: Graft-versus-host disease reduces lymph node display of tissue-restricted self-antigens and promotes autoimmunity
Source: J Clin Invest. 2020 Mar 9;130(4):1896–911. doi: 10.1172/JCI133102 (PMC7108931; doi:10.1172/JCI133102)
Supplement: Supplemental data [file jci-130-133102-s008.pdf]

Supplemental material

Supplementary Figure 1

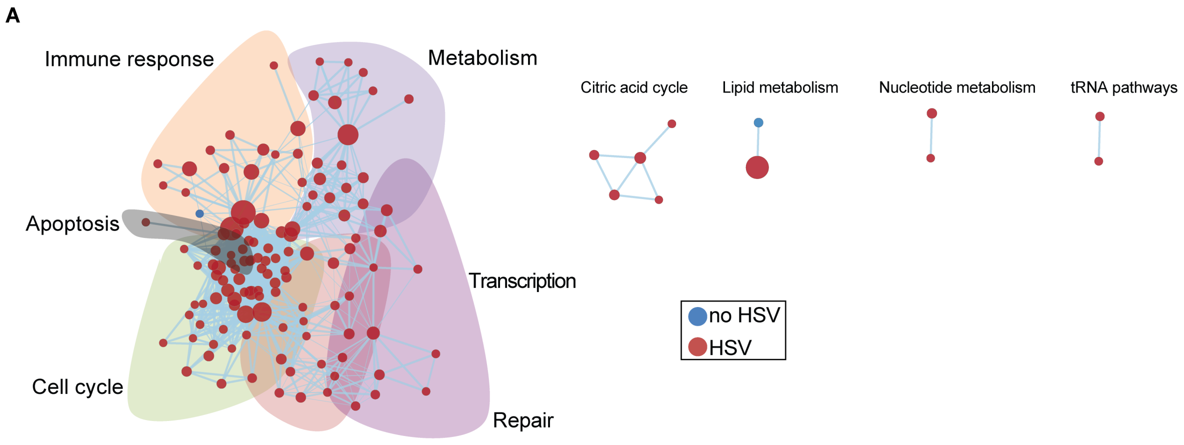

Supplementary Figure 1. Network visualisation of differentially up-regulated REACTOME pathways in FRC from HSV-1 infected hosts using EnrichmentMap.

(A) Enriched REACTOME pathways are depicted by red and blue nodes, where blue represents significant up-regulation in non-infected FRC and red represents significant up-regulation in HSV-1-infected FRC. Node size is proportional to the number of genes in each node, line thickness indicates the overlap of genes between nodes, and the theme of genes in each cluster is specified.

Supplementary Figure 2. FRC depletion in HY-mismatched GVHD model according to donor T cell function and subset or to treatment.

(A) Immunohistochemistry and confocal microscope analysis was performed on frozen LN sections taken from transplant recipients 7 days after BMT (i-iii, TCDBM; iv-vi, TCDBM+T). gp38 (podoplanin, red) and CD31 (green) was used to denote different LN stroma subsets. Scale bars = 100 µm. **(B)** Absolute numbers and frequencies of LN FRC in recipients without acute GVHD (TCDBM) compared with recipients with acute GVHD that have either received TCDBM+ Mh $CD8^+$ T cells or TCDBM+ perforin-deficient *($pfp^{-/-}$)* Mh $CD8^+$ T cells at 3 weeks following F→M BMT (data derived from 6 independent experiments). **(C)** Absolute FRC numbers and frequencies 28 days after F→M BMT and 21 days following either Marilyn $CD4^+$ or MataHari $CD8^+$ T cell transfer (both transferred on day 7 post BMT). Controls received TCDBM alone (data derived from 3 independent experiments). **(D)** GVHD score of TCDBM, TCDBM+T and TCDBM+T recipients that received anti-CD8α depleting antibody on days 5 and 19 post-transplant (left) and frequency of donor CD8 T cells in spleens of acute $GVHD^+$ recipients +/- anti-CD8 antibody (right). Data represent mean±SEM. **p<0.05, **p<0.01, ***p<0.001, Mann-Whitney U-test (2D, right), Kruskal-Wallis ANOVA (2B,C,D left).*

Supplementary Figure 2

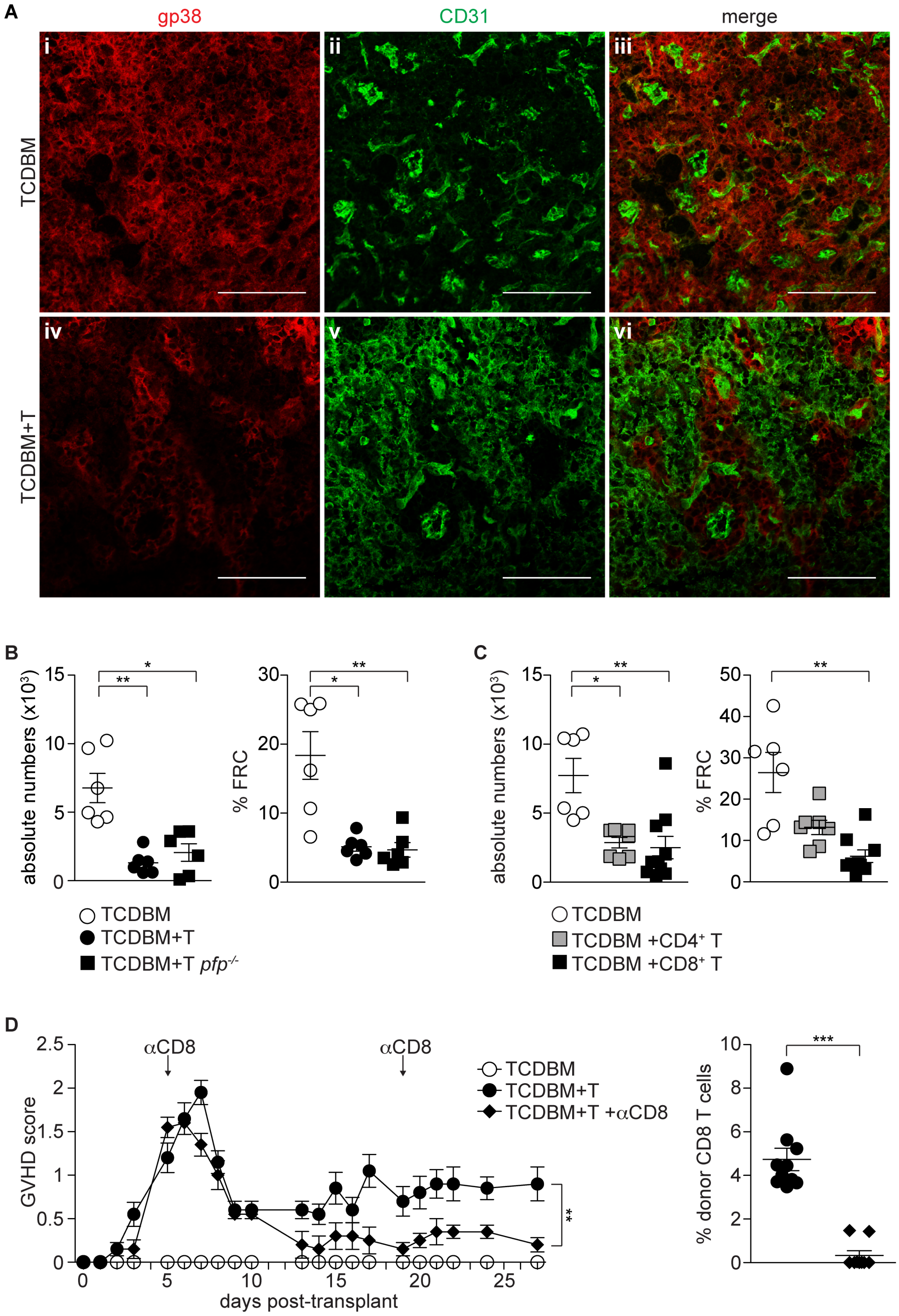

Supplementary Figure 3. FRC depletion in multiple miHA-mismatched GVHD model according to donor T cell function and subset or to treatment.

(A) Weight change in recipient mice after TCDBM, TCDBM+T or TCDBM+T_{CD62L-} transfer is shown as percentage of initial body weight (=time point of transplant, data derived from 4 independent experiments). **(B)** GVHD score of TCDBM, TCDBM+T and TCDBM+T + post-transplant cyclophosphamide (PTCy) recipients (left) and frequencies of donor CD4 and CD8 T cells in spleens (right). PTCy was administered on days 3 and 4 post-transplant (25 mg/kg/day). **(C)** GVHD score (left) and survival curve (middle) of TCDBM and TCDBM+T with or without dexamethasone (Dex) treatment. Right: Percentage of donor T cells at 3 weeks after BMT in $GVHD^+$ mice that were either treated with Dex (0.3 mg/kg/day) or PBS starting at day 5 after BMT (data derived from 2 independent experiments). **(D)** Frequencies and absolute numbers of FRC in untransplanted mice that were either treated with Dex or PBS for 14 days before take-down. Data represent mean±SEM. **p<0.05, **p<0.01, ***p<0.001, Mann-Whitney U-test (3C right,D), Kruskal-Wallis ANOVA (3A,B,C).*

Supplementary Figure 3

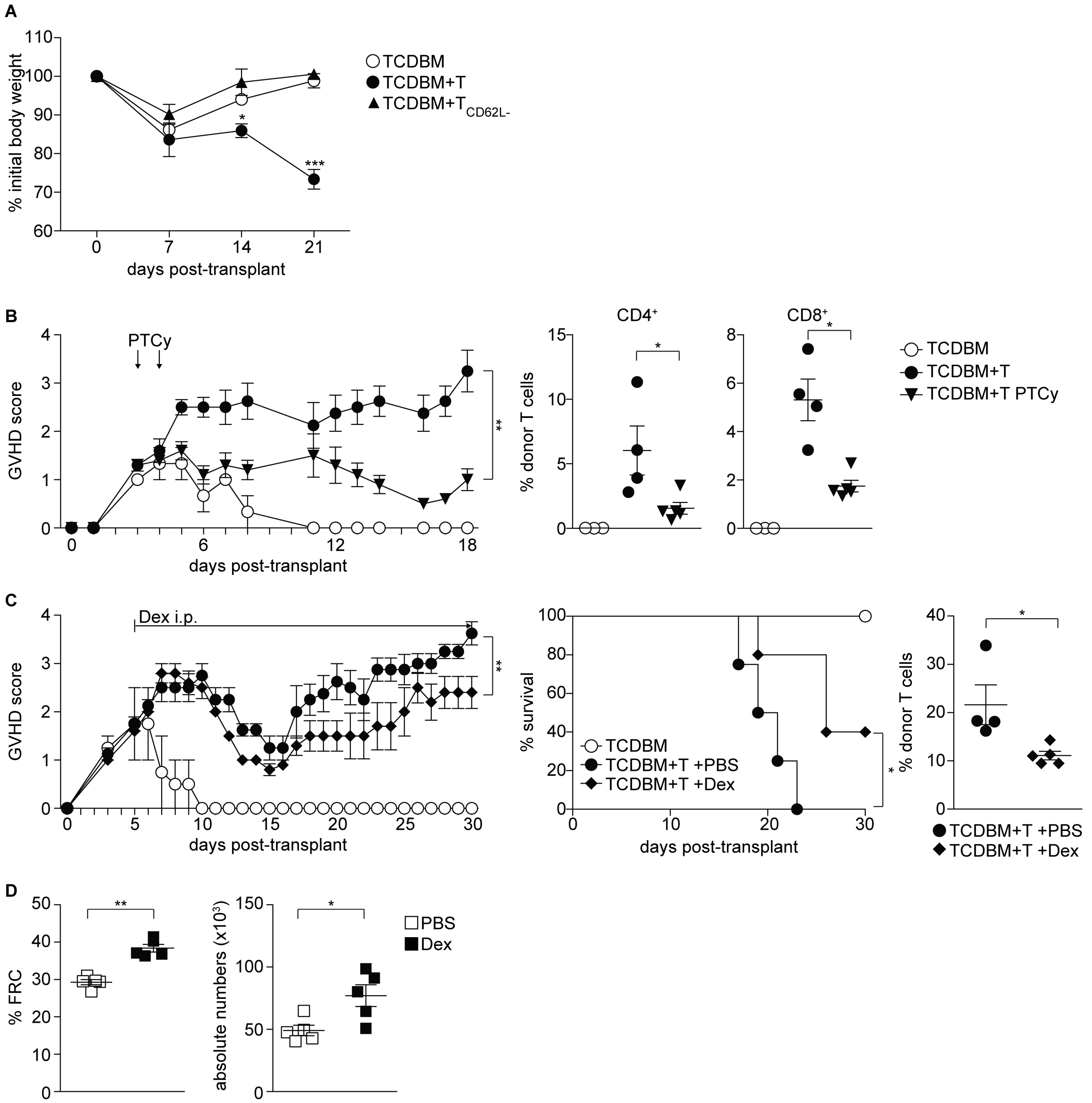

Supplementary Figure 4. Stromal reorganisation following BMT.

(A) Gating strategy used to identify $lin^{-}CD117^{+}CD127^{+}Ror\gamma t^{+}$ LTi cells (top). Lineage markers: CD3, CD19, NK1.1, LY6G, F4/80. Representative flow cytometry plots depicting surface expression of CD4, NKp46 and CCR6 among $lin^{-}CD117^{+}CD127^{+}Ror\gamma t^{+}$ cells (bottom). **(B)** Absolute numbers of host and donor LTi cells were evaluated at indicated time points following B6→129 BMT. Dotted line indicates mean absolute numbers of LTi cells in untreated mice (data derived from 3 independent experiments). **(C)** Surface expression of VCAM1 and ICAM1 on $CD45^{-}CD31^{-}gp38^{+}$ LN stromal cells in un-transplanted controls (grey) and recipients of TCDBM alone (blue) or TCDBM+T (red) at indicated time points following B6→129 BMT. Summary data depicting MFI of VCAM1 among $CD45^{-}CD31^{-}gp38^{+}$ population in TCDBM versus TCDBM+T recipients are shown below the respective flow cytometry plots (data derived from 6 independent experiments). Data represent mean±SEM. **p<0.05, **p<0.01, Mann-Whitney U-test.*

Supplementary Figure 4

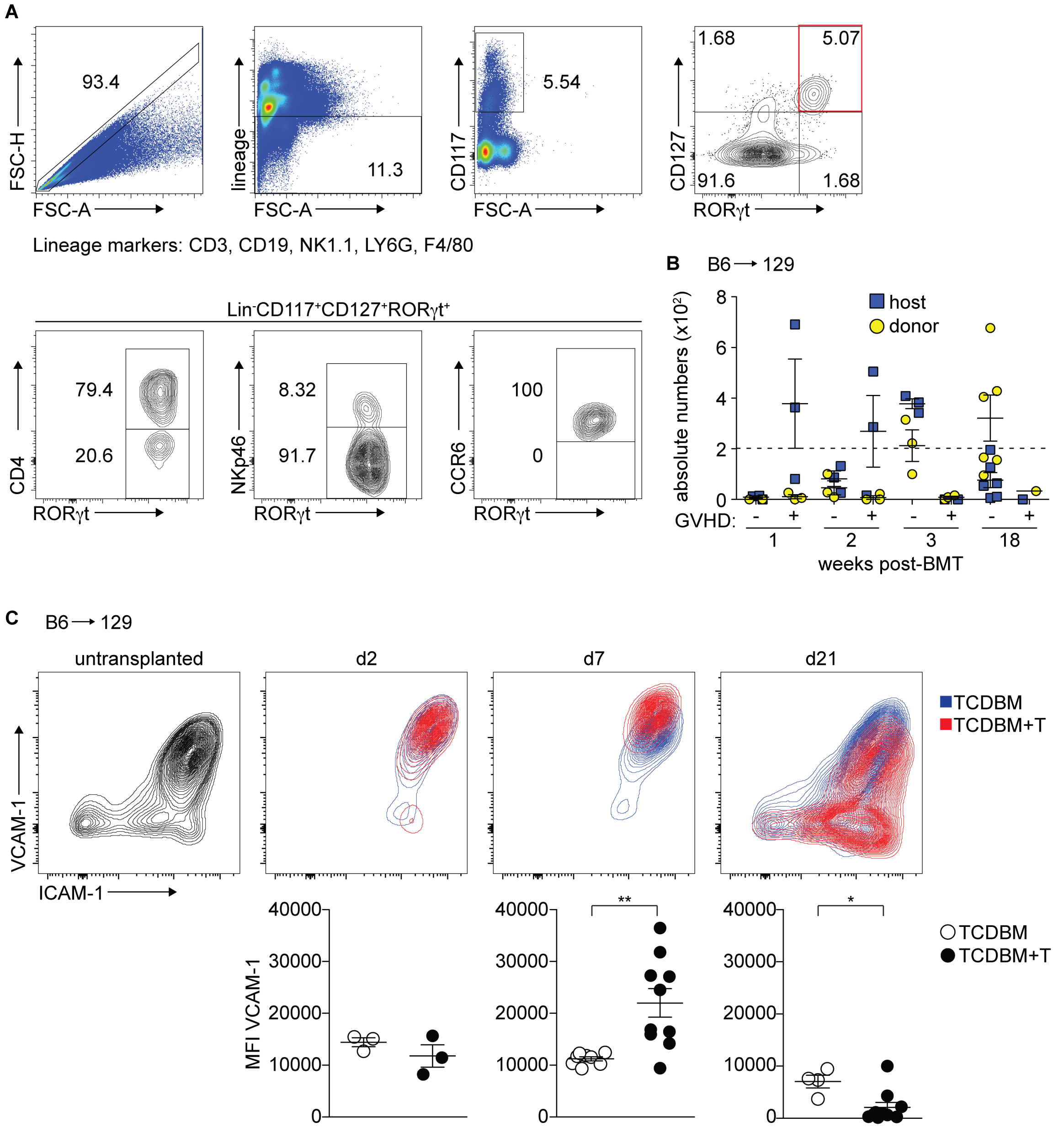

Supplementary Figure 5. OT-I T cell responses following BMT in OVA-positive or -negative recipient mice.

(A) Absolute FRC numbers (left) and OVA expression measured by qPCR (right) on day 14 after BMT in iFABPtOVA recipients with or without acute GVHD (data derived from 3 independent experiments). **(B)** Percentage and absolute numbers of OT-I T cells in LN in TCDBM and TCDBM+T iFABPtOVA recipients (left). Frequency and absolute numbers of IFNγ^+ $CD8^+CD45.1^+$ OT-I T cells in LN in absence and presence of acute GVHD is summarised (right) (data derived from 7-8 independent experiments) **(C)** Flow cytometry plots depict surface expression of CD45.1 and Thy1.1 among $CD8^+$ T cells (OT-I T cells- $CD45.1^+Thy1.1^-$; Mh T cells-$CD45.1^+Thy1.1^+$). Frequencies of OT-I T cells among total live cells in MLN and IEL isolated from OVA-negative B6 recipients with or without acute GVHD are summarised in dot plots (right panel) (data derived from 3 independent experiments). **(D)** Donor-derived DC were depleted by DT administration in hosts transplanted with CD11cDTR BM. Flow cytometry plots depict CD11b and CD11c surface expression among $MHCII^+$ splenocytes (data representative of 3 independent experiments). Data represent mean±SEM. **p<0.05, **p<0.01, ***p<0.001, Mann-Whitney U-test.*

Supplementary Figure 5

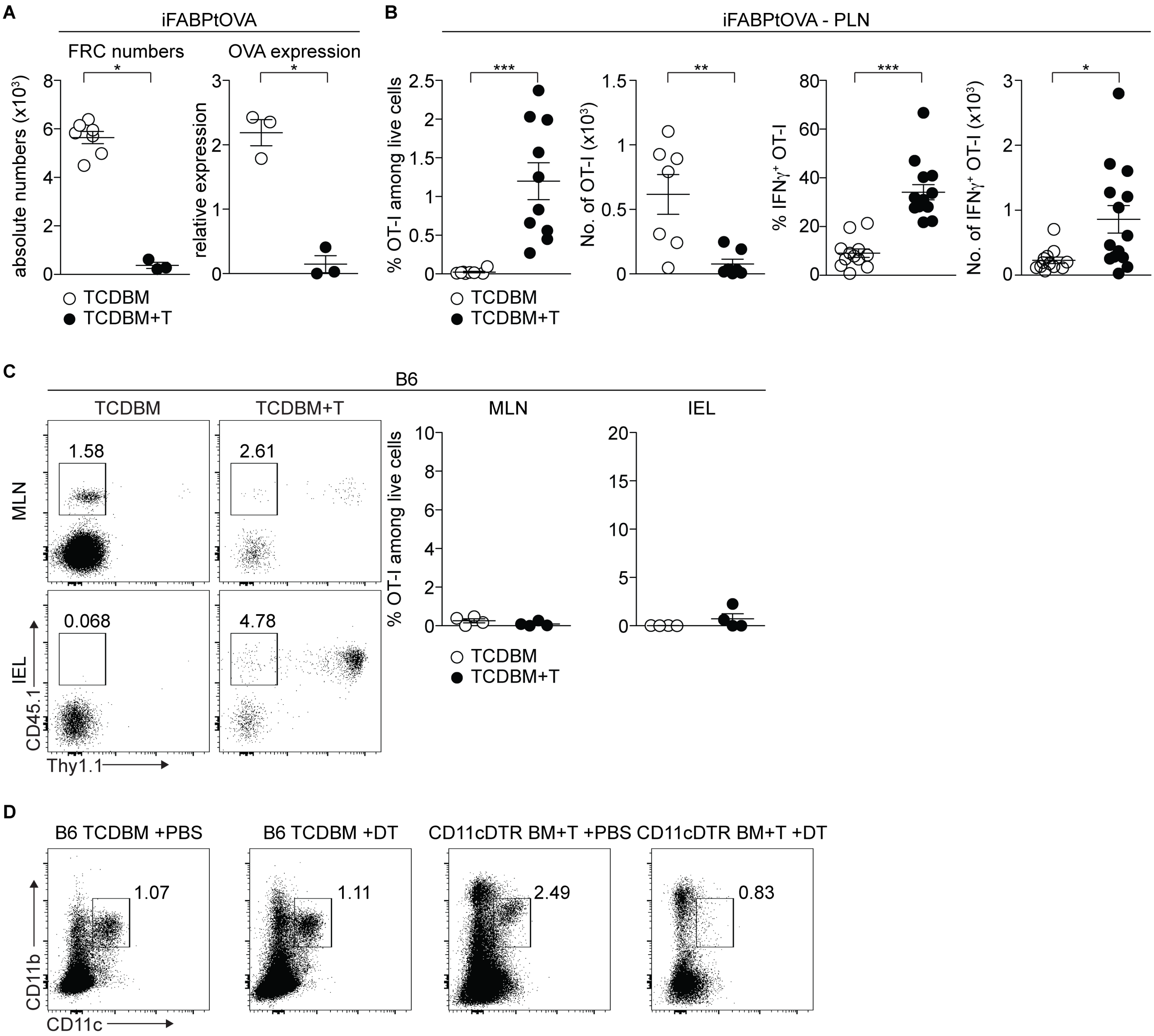

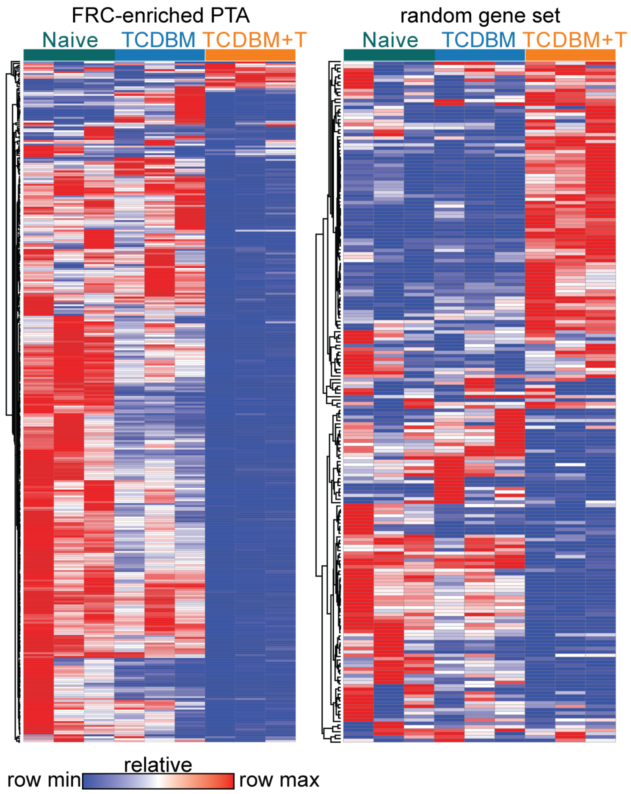

Figure S6. Expression of FRC-enriched PTA and random gene set in acute GVHD.

Relative expression of FRC-enriched PTA in FRC isolated from un-transplanted controls, TCDBM or TCDBM+T recipients at day 7 post-BMT following F→M BMT is shown as a heatmap (left panel). The expression values of a randomly selected gene set are depicted as a control (right panel).

Supplementary Table 1: FRC-specific PTA

Gene Symbol	log2(FC) FRC vs BEC	p-value FRC vs BEC	log2(FC) FRC vs LEC	p-value FRC vs LEC	log2(FC) TCDBM+T vs TCDBM	p-value	FDR-adjusted p-value
Mmp11	1.84185	3.22159E-05	1.38719825	0.000230797	-7.28906	1.2681E-12	7.71047E-11
Art4	2.1008305	0.000267676	1.50371125	0.000816867	-7.28086	3.50405E-08	6.76994E-07
Pamr1	5.5186775	6.25076E-08	5.75203175	1.8781E-08	-7.02606	8.88178E-16	1.2401E-13
Crym	2.49718825	5.61225E-06	2.66882725	7.71745E-06	-6.6937	0.000374034	0.00225475
Camk1g	1.382228	4.97257E-05	1.608473	7.09459E-05	-6.57696	0.00813865	0.030595
St6galnac5	2.1887265	9.27986E-05	2.36828775	3.64275E-05	-6.56159	0.000436641	0.00257856
Vsnl1	3.02630925	0.000751317	3.13883925	0.000215383	-6.49544	0.00465497	0.0192053
Rgma	1.66125775	2.02626E-05	1.3170015	0.000170366	-6.23247	0.00279953	0.0125057
Itgbl1	1.64742	0.000273615	1.715825	0.000765575	-6.18713	1.50223E-06	1.84467E-05
Saa3	1.9345155	0.041583926	2.197524	0.031256913	-6.16906	2.0528E-12	1.20404E-10
Evc	2.47248975	5.29578E-07	1.64500725	7.91898E-06	-5.72321	1.6183E-07	2.59704E-06
Fgf14	1.67367875	0.000770557	0.884191	0.006962998	-5.67577	0.0425627	0.118344
Podn	2.88293925	4.91795E-07	2.885481	1.26127E-05	-5.61286	5.71395E-10	1.77222E-08
Dpyd	3.600878001	0.000210771	0.03782325	0.460324632	-5.56294	1.15145E-07	1.93049E-06
Slco2b1	0.313975	0.013692353	3.2271525	2.16853E-05	-5.55569	6.77236E-14	5.83859E-12
Igj	1.958651	0.022747815	1.87876975	0.024749643	-5.50231	1.3942E-07	2.28697E-06
Cyp2j9	1.162134	0.001186655	3.72638925	3.13335E-05	-5.36583	6.00132E-08	1.08816E-06
Rbm46	1.59391925	0.000063143	1.69108825	0.000178997	-5.29543	0.00316797	0.0138898
Fgfr4	1.58135225	0.000220556	2.078901	3.32734E-06	-5.27094	0.000664583	0.00368189
Gpnmb	3.24086425	0.000156385	2.5945905	0.000328837	-5.25136	0	0
Nfasc	2.13819275	1.47794E-06	2.217069	5.43405E-07	-5.21531	1.59662E-05	0.000147687
Cd14	1.1376065	3.37599E-06	1.74100075	0.00352894	-5.21216	3.83061E-10	1.2597E-08
Cpxm2	2.4118965	0.000319341	2.7325735	0.000447552	-5.19499	3.23162E-05	0.000271383
Ugt3a2	2.50929325	0.000408713	2.662067	0.000400125	-5.1789	8.54957E-05	0.000629052
Abca6	3.79270125	3.27161E-06	3.86140875	9.04326E-06	-5.12023	0.00495747	0.0202718
Robo2	1.40744975	0.002517521	3.177628	6.51719E-06	-5.09287	9.20053E-07	1.19041E-05
Npy1r	3.73892175	2.22148E-05	5.18335375	2.92896E-06	-5.04969	6.20491E-09	1.46613E-07
Serpinb8	1.92330025	6.11961E-05	3.114065	1.53759E-06	-5.04282	0.000754057	0.00410411
Il7	4.156331	2.91041E-05	1.51486425	0.000205698	-4.98064	7.43849E-14	6.2695E-12
Dmc1	1.824749	2.12811E-05	1.843156	0.000185741	-4.94834	0.00700025	0.0270336
4833424O15Rik	3.33865775	5.59151E-06	3.372567	0.000027723	-4.94326	0.00454931	0.0188483
Agmo	2.03215025	2.16886E-05	3.1320005	7.43296E-05	-4.94033	7.71441E-08	1.36186E-06
Col6a4	2.38850175	0.000012689	2.9007355	1.49526E-07	-4.93516	2.00136E-10	7.03325E-09
Gxylt2	3.76147675	2.50362E-05	-0.0856195	0.29762633	-4.92243	2.46585E-09	6.4923E-08
Thbs2	4.634388	5.75431E-08	4.03174625	1.35441E-06	-4.90627	0	0
Cxcl2	0.9327085	0.006453658	2.09456825	0.000173574	-4.87225	2.15093E-10	7.48277E-09
Cldn1	6.0734645	1.45575E-06	5.1591995	8.87838E-06	-4.86372	2.39456E-09	6.3285E-08
Gli3	1.40055025	3.71653E-05	2.185985	4.04385E-06	-4.85614	2.01347E-05	0.000180023
Cpeb1	2.74191475	1.24537E-07	2.575463	9.01876E-09	-4.81848	6.07364E-08	1.0998E-06
Abca8b	2.460699	4.67628E-06	2.34007675	7.70216E-06	-4.81462	1.62141E-05	0.000149714
Msc	3.10612125	1.77557E-06	2.3546705	7.28646E-06	-4.80232	2.06026E-11	9.52079E-10
Zmynd15	3.30150125	9.86522E-06	1.44430025	0.000141963	-4.79864	0.000107424	0.000766328
Lpar4	0.18116075	0.312741619	2.6506165	0.000677273	-4.7736	0.000746553	0.00406861
Nos1ap	1.28565725	0.000457507	1.9206045	0.000333491	-4.74044	0.00548514	0.022049
Kcnma1	3.06794775	1.02307E-07	3.234119	2.38828E-06	-4.72081	0.000024254	0.000211651
Unc5c	2.92988975	5.51265E-07	0.997852	0.000237607	-4.6889	8.74212E-07	1.13785E-05
Meox2	2.25810975	6.45555E-05	0.62732175	0.041327269	-4.68619	4.60159E-11	1.90438E-09
Hs3st3a1	3.60736475	0.000139237	1.617592	0.001377748	-4.67732	6.47915E-07	8.72895E-06
Larp6	3.045898	7.75348E-07	2.8497945	1.6811E-06	-4.67618	2.00885E-08	4.13825E-07
Clip3	4.08589975	5.01894E-07	3.52410075	1.99813E-06	-4.66833	2.34917E-11	1.06001E-09
Ctsk	4.643297	1.80307E-08	2.26049675	7.44795E-06	-4.61501	1.32547E-11	6.41362E-10
Adamts15	1.505902	0.000010499	1.88613075	2.43321E-06	-4.61341	2.32703E-13	1.70189E-11
Apoc1	2.28237375	0.000419161	2.18998875	0.000837434	-4.60875	0.000045565	0.000364978
Fmo2	-0.68478475	0.042077468	2.8248135	0.000723809	-4.58534	2.87168E-09	7.42411E-08
Orm2	1.68387375	0.000987842	1.88234775	0.000978318	-4.56594	0.000869149	0.00463859
Irs3	1.29667625	0.0001148	1.6325815	4.31841E-06	-4.56031	0.000305818	0.00189789
Thbs4	1.85708425	0.010969793	1.11472775	0.041480836	-4.55506	6.98478E-06	7.14467E-05
Rtn2	1.97337075	3.52507E-05	1.72778775	0.00019707	-4.53236	0.0491244	0.132156
Tbx15	4.41863325	5.68999E-07	4.66030675	4.29281E-08	-4.52799	1.02758E-08	2.28358E-07
Il34	1.402583	0.000235728	2.40861975	1.37985E-05	-4.52642	0.000229764	0.00148733
Nr1d1	0.16633825	0.35024344	1.5886605	0.008107445	-4.51466	1.40288E-12	8.49263E-11
Csmd1	3.24343375	1.6223E-08	3.829135	2.32325E-09	-4.50632	4.71471E-07	6.62072E-06
Vwa1	-1.360478	0.000044177	2.25088825	5.50663E-06	-4.48716	1.69861E-10	6.07747E-09
Olfml1	4.22141875	3.12581E-06	4.34878975	7.4898E-07	-4.46228	1.27159E-09	3.60918E-08
Plekhh2	3.94928825	1.82036E-06	4.122882	6.02776E-05	-4.44137	2.29916E-08	4.65054E-07
Cyp2d22	-0.660909501	0.002547729	1.953035	9.38492E-06	-4.42319	2.61484E-09	6.84114E-08
Scn7a	5.702059	0.000034461	5.89185425	1.10789E-05	-4.41734	1.7748E-12	1.0548E-10
Lrrc4c	1.372211	0.000213348	1.684747	8.63568E-05	-4.41452	0.000628178	0.00350879
Colec12	3.503097751	1.46712E-07	0.99443625	0.000373213	-4.40289	1.11022E-15	1.49963E-13
9930013L23Rik	1.9970545	1.81674E-06	1.9762295	3.26587E-06	-4.37029	0.00126954	0.00642017
Ccdc3	2.121856	1.83039E-05	-0.668768501	0.015851238	-4.36966	7.51904E-07	9.98078E-06
Adcyap1r1	4.2882315	2.04295E-08	4.770883	1.58351E-09	-4.36356	3.27081E-11	1.40991E-09
Kcnq4	1.871557	7.39106E-07	2.38600575	3.14795E-07	-4.35728	0.00062502	0.00349304
Gabrg3	2.12280875	7.88896E-05	2.1617825	9.15387E-05	-4.35509	0.00633976	0.0249215
Mrc2	3.179531	2.02664E-07	3.47328275	6.62292E-07	-4.35478	4.02987E-10	1.31687E-08
Slc36a2	3.5121505	2.52809E-05	3.67185725	3.37979E-05	-4.34336	3.41624E-09	8.63809E-08
Grem1	4.92369225	1.11589E-05	5.053156	1.00931E-06	-4.31278	4.41043E-09	1.0867E-07
Mustn1	1.57647725	0.001897466	2.32823875	0.000137232	-4.31135	1.76836E-11	8.3047E-10
Svep1	5.346474	2.71778E-08	5.41056925	8.2548E-08	-4.30843	3.93979E-10	1.29337E-08
Cc2d2a	1.72070225	2.56355E-05	1.362745	7.89055E-05	-4.2847	3.68648E-06	4.07439E-05
Gfra2	4.34841975	1.34642E-07	3.599204	5.61544E-08	-4.28143	1.3753E-08	0.000000296
Tbx18	3.1784455	1.51121E-07	3.43902975	3.12981E-07	-4.25348	0.00020865	0.00136925
Gli2	2.027148	1.94545E-06	2.1134375	4.36013E-06	-4.23471	3.63711E-05	0.000300386
Myo16	3.165056	3.76156E-08	2.905914	5.51465E-06	-4.22454	0.000725512	0.00397011
Hmgcs2	3.12090775	0.000326426	5.895215	1.84152E-09	-4.20335	3.10418E-13	2.21176E-11

Akr1c19	2.03707375	0.001316908	2.19986225	0.00136103	-4.20108	0.00811414	0.030527
Wdr86	2.62416525	0.000011395	2.78560675	7.4338E-06	-4.19562	8.25939E-06	8.26696E-05
Adamts20	2.06238925	8.36913E-05	2.212804	2.67549E-05	-4.18165	0.00311873	0.0137057
Slc7a11	4.6073055	1.14947E-05	3.80946275	2.19531E-05	-4.15989	1.58793E-09	4.37975E-08
Synpo2	2.33311925	6.17752E-05	3.40189575	8.1167E-08	-4.15581	2.0301E-10	7.12746E-09
Abcd2	2.311563	0.000048184	0.925019	0.004004879	-4.15566	1.05587E-07	1.78859E-06
Ntng1	1.967852	0.000947016	2.07470225	0.001206819	-4.14411	0.206727	0.396554
Avpr1a	2.69777525	5.53088E-06	2.37303425	2.17837E-06	-4.1402	4.36314E-06	4.71051E-05
Cacna1c	2.24243725	6.95405E-07	2.437894	3.05792E-08	-4.13109	0.0345573	0.100284
Rai2	2.13624275	1.31697E-06	2.0488775	1.38694E-06	-4.11786	0.000144743	0.000995557
Stc2	1.71557125	0.000330317	0.13961775	0.273017466	-4.08919	0.00398127	0.0168671
Dsc3	7.049988	5.70968E-09	6.29068325	1.15805E-05	-4.0865	4.35385E-12	2.35217E-10
Pla2r1	2.3374115	2.73083E-05	1.668364	0.000284466	-4.07209	1.96256E-08	4.05329E-07
Pappa	1.645558	0.001129985	1.61474175	0.000821637	-4.03721	0.000251061	0.00160219
Plce1	2.579545	1.16752E-06	-0.037293499	0.386640996	-4.02356	4.64863E-05	0.000371639
Plscr2	-2.249755503	8.70611E-05	2.48626075	1.73628E-05	-4.02236	2.49855E-05	0.000217029
Rasl12	2.40041175	1.54632E-05	3.44877425	5.26709E-06	-4.01639	7.63302E-06	7.72957E-05
Mxra7	1.3520315	3.44476E-05	2.17847525	7.10863E-06	-3.98493	1.81665E-09	4.94309E-08
Sec16b	1.89812175	2.01741E-05	1.655873	1.23114E-05	-3.98013	0.000686676	0.00378709
Sulf1	0.77752	0.010032462	1.853816	0.000266461	-3.96802	1.26736E-10	4.70922E-09
Kazn	1.59183925	2.25704E-05	2.32571	2.50882E-06	-3.95923	2.60729E-05	0.000225248
Adamtsl1	1.7581915	0.000358321	0.73338225	0.008836849	-3.9584	3.57321E-05	0.000295815
Tmeff2	2.4102365	0.000504546	2.39688475	0.000185833	-3.95544	2.8751E-08	5.67465E-07
Ggt5	1.990379	2.11896E-05	3.729564	4.77168E-07	-3.94094	5.17275E-12	2.75005E-10
Pcdh19	0.28713075	0.1146819	2.361914	1.56197E-05	-3.92995	0.0183237	0.0598777
Pcdh15	4.296555	1.45051E-06	2.504662	0.002921309	-3.92722	0.000451353	0.00265252
Mrvi1	2.32895775	2.45627E-05	2.936768	2.92231E-07	-3.91835	4.79061E-07	6.71592E-06
Ccdc8	2.01322125	1.01577E-06	2.10502975	9.78324E-05	-3.90096	0.000635683	0.003544
Vtn	5.5303665	1.54892E-05	6.38482075	2.55918E-05	-3.88303	3.17746E-13	2.24468E-11
AI464131	1.9054015	7.9391E-06	2.080264	4.66122E-05	-3.87373	1.94669E-06	2.32616E-05
Dtx4	3.707797	1.11341E-07	4.21372425	4.75219E-06	-3.86348	1.02363E-13	8.24227E-12
Prr16	2.23866275	2.72349E-06	1.292045	0.000143879	-3.86023	2.78952E-07	4.16549E-06
Pth1r	1.60848225	0.000290199	1.6946835	7.13437E-05	-3.85751	3.35265E-06	3.74536E-05
Nxph1	-0.59237	0.070428561	1.649701	0.000318882	-3.84323	0.000781575	0.00423388
Pcdh18	2.3835185	5.60526E-06	3.20861575	8.36962E-07	-3.83563	4.19163E-05	0.000339622
Gprasp2	2.37156625	2.43343E-06	1.5471375	3.65188E-05	-3.82752	0.0133427	0.0459623
Gpc6	4.39239825	7.88257E-09	4.31571975	5.2175E-07	-3.78435	6.3224E-09	1.48964E-07
Cpz	3.349586001	1.74882E-05	3.414561	4.21965E-07	-3.7766	0.000301312	0.00187357
Lphn3	2.006604	4.26865E-06	0.86991675	0.000430284	-3.76544	0.00165722	0.00803102
Atp1b2	1.58366175	4.89832E-05	2.44063275	2.20967E-06	-3.75584	2.52535E-06	2.93091E-05
Serpine1	2.87904375	0.000344525	0.48693425	0.181382485	-3.75184	0.00512875	0.0208557
Rnf150	3.14709425	1.48989E-06	3.13757075	8.78971E-06	-3.73188	6.78434E-07	9.09874E-06
Stmn2	3.1727575	9.53286E-06	4.76697825	7.22095E-07	-3.72809	7.29033E-09	1.68233E-07
Cml3	2.58612775	8.74395E-05	2.702029	0.000052103	-3.72319	0.000862387	0.00460784
Ngfr	2.35628675	0.000507184	1.64934375	0.000844794	-3.72213	0.00208076	0.00972662
Pthlh	2.4117465	2.61018E-06	1.3065975	0.000106844	-3.71793	1.89164E-05	0.000170675
Fap	4.59964375	3.59605E-07	4.8431225	1.29066E-07	-3.70571	4.04763E-08	7.65726E-07
Mark1	2.2464035	2.38452E-07	2.75927325	3.87224E-08	-3.69447	2.73359E-07	4.09357E-06
Gpr176	3.94736125	1.55848E-08	4.109118	5.12569E-07	-3.68314	9.41465E-09	2.11373E-07
Bst1	2.197856	4.91053E-05	2.13532625	5.07493E-05	-3.6793	1.50584E-10	5.47274E-09
5430435G22Rik	3.173897	9.58061E-06	3.3057765	1.18851E-05	-3.67742	5.24842E-06	5.55775E-05
Tmem35	2.013254	0.000283591	2.2964815	8.91159E-05	-3.67488	0.0150921	0.0509933
Rab30	3.09652425	2.95049E-06	1.72382875	7.48163E-05	-3.6742	0.000904674	0.00480039
Aspn	1.79782675	0.000168159	3.29217875	1.30897E-06	-3.66859	0.0245295	0.0758474
Hgf	4.16431775	8.35209E-06	3.98039525	4.34403E-05	-3.6582	7.38679E-06	0.000075059
Spib	4.035876	1.29426E-07	0.93722375	0.001889534	-3.63644	3.04829E-11	1.32641E-09
Srpx2	3.899632	3.75847E-08	4.12494975	1.27425E-09	-3.63359	0.00011536	0.000816062
C1qtnf7	3.07435025	7.46408E-09	-1.22837425	0.00606031	-3.62126	0.000421991	0.00250381
Adm	1.8112765	0.000447722	0.47633725	0.060151063	-3.60416	1.91611E-06	2.29342E-05
Sned1	2.08030775	0.000539725	0.895596	0.007721556	-3.59625	3.10638E-10	1.04051E-08
Mmp3	5.79451325	1.78218E-05	5.90742425	1.21424E-05	-3.59061	7.73936E-09	1.77902E-07
Nkd1	2.701583	2.90791E-05	2.330552	1.38994E-06	-3.58345	2.36555E-06	2.76869E-05
Ildr2	3.022163	2.1865E-07	3.2093945	1.22396E-05	-3.5695	5.35341E-09	1.29217E-07
Slc43a1	2.537693	9.1811E-06	2.21279825	0.000019745	-3.56011	8.11049E-07	1.06736E-05
Cpxm1	6.444203	3.85617E-07	3.60971875	2.21808E-05	-3.55816	1.30318E-05	0.000123747
Lhx8	3.44883	2.38235E-06	3.568181251	4.26126E-05	-3.55232	2.20376E-08	4.48308E-07
Rhbdl3	1.9594525	1.59382E-06	1.838534	8.13503E-06	-3.55102	3.04396E-07	4.50007E-06
Msx1	1.1099345	2.63111E-05	2.65929775	1.04789E-07	-3.5369	0.0337168	0.0983033
Ptgs2	4.5643965	0.000038264	3.400832	8.63191E-05	-3.53369	1.08886E-09	3.14654E-08
Tusc5	2.116556	0.000270389	2.95026125	3.02084E-06	-3.53343	0.00125736	0.00637098
Sh3pxd2b	3.04149875	1.99895E-08	2.68885775	4.47934E-05	-3.53276	3.53655E-06	3.92646E-05
Mmp19	3.5478025	6.2944E-07	3.59549475	3.72969E-07	-3.52334	1.20317E-06	1.51466E-05
Tnfsf13b	5.15154475	2.55331E-08	5.0477925	2.02566E-07	-3.50333	4.47367E-10	1.44145E-08
Ntrk1	3.516506999	7.35775E-06	3.335945501	2.94476E-06	-3.50264	4.5862E-11	1.8999E-09
Kcnj8	3.196571	0.000010212	2.7150795	1.04202E-05	-3.47881	2.51887E-09	6.61509E-08
Nbl1	2.1684805	0.000377222	2.63735875	5.33062E-05	-3.47191	1.69354E-08	3.54864E-07
Adamtsl3	2.25209675	4.7772E-06	2.027163	2.34247E-05	-3.46673	8.92383E-05	0.000653112
Arntl2	1.33273475	4.84465E-05	1.72836475	0.000254171	-3.46406	0.00053218	0.00305488
Serpina3c	2.83309675	2.72731E-06	3.298846	4.02492E-05	-3.45148	5.12489E-08	9.46632E-07
Rtn1	3.53522275	2.78259E-07	3.16935675	5.38242E-07	-3.44833	0.000351843	0.00214217
Arhgap20	2.019923	5.39143E-05	2.82198	6.25325E-05	-3.44575	0.000590582	0.00333473
Pde1a	1.9759305	1.20759E-05	2.3181645	4.71098E-05	-3.43962	0.00300739	0.0132897
Adc	2.74120875	9.74494E-06	1.69648275	1.24737E-05	-3.42813	4.6762E-07	6.57777E-06
Gucy1b3	1.8206695	0.001269398	1.72428575	0.000360212	-3.42361	0.000131045	0.000913764
Anpep	2.70584375	3.90227E-05	-0.1624975	0.241481927	-3.41743	2.91109E-06	3.32188E-05
Gm266	1.7908485	7.91551E-06	1.9291805	2.42647E-06	-3.41623	0.0216671	0.0686816
Tspan11	1.7365595	4.46519E-05	0.7154325	0.002468466	-3.40851	0.000138961	0.000960405
Cacng7	2.471427	6.38843E-07	2.73306775	2.4156E-07	-3.40744	0.303277	1

Naalad2	4.41197975	2.68605E-08	4.86495875	2.85743E-07	-3.39678	1.00893E-07	1.71962E-06
Gstm7	1.17175925	0.000531939	1.64242625	0.000256523	-3.39391	0.00368271	0.015791
BC005764	2.080852	0.000349784	2.3525865	7.99592E-05	-3.39098	0.920126	0.97244
Cyp7b1	1.70225625	9.81819E-05	0.5497525	0.004723096	-3.38846	3.11092E-06	3.51129E-05
Ccl19	6.31466525	2.62059E-08	4.921961	0.000101603	-3.36438	0.0141607	0.0482988
Slc45a3	2.11461	4.81229E-05	-2.26863775	2.15375E-06	-3.35837	1.12835E-05	0.00010884
Itga8	1.831936	1.04544E-05	0.6085325	0.002917143	-3.35702	0.00049606	0.0028754
Pla2g2d	2.808317	0.000367764	2.2979585	0.000329662	-3.33438	4.72524E-08	8.83436E-07
Foxs1	2.08644175	1.67881E-05	2.5217425	5.78022E-06	-3.32014	0.0002709	0.00171088
Chrdl1	5.5338415	7.72429E-07	4.98888775	7.96932E-07	-3.31676	4.82735E-09	1.17892E-07
Gabra3	4.22437675	0.000119562	4.298875	6.80189E-05	-3.29195	0.00021403	0.00139946
Syt12	1.8604285	1.72662E-05	1.99075175	7.22047E-06	-3.28874	0.0607913	0.156889
Akr1cl	3.0660785	2.57923E-05	3.34097025	9.88292E-05	-3.25316	0.00105323	0.00546351
Nckap5	1.59222875	2.46006E-05	0.813787	0.010015645	-3.23837	0.00714405	0.0275095
C1qtnf1	-0.014063	0.462203486	3.17910425	2.96973E-06	-3.23641	7.69118E-06	7.77329E-05
Osr1	3.347292749	2.95329E-06	3.0997665	8.37396E-05	-3.23271	6.29994E-08	1.13684E-06
Arhgap6	2.375876	2.33459E-06	2.24222925	3.52531E-06	-3.22418	0.00195568	0.00924196
Adamts2	3.148113	6.36833E-08	3.223011	4.06929E-08	-3.21986	2.75099E-09	7.15222E-08
Frzb	1.70073275	0.007231727	1.69771975	0.005934443	-3.21903	0.000569163	0.00323405
Gpr64	3.0158795	0.000227437	3.03515025	0.000179657	-3.20949	0.00728622	0.0279686
Slit3	4.05080175	9.15373E-08	4.04194625	2.76348E-07	-3.18291	6.66298E-11	2.65307E-09
Spic	3.56777425	2.33982E-06	2.792626	3.70727E-05	-3.17194	7.01939E-06	7.17123E-05
Glt8d2	2.75468225	1.40763E-06	2.59620675	4.96069E-06	-3.15647	0.459091	0.673452
Tiam2	1.79055175	1.45767E-06	0.465444	0.006622183	-3.13042	0.0298132	0.0889484
Pcolce2	4.42659075	0.000103195	4.35115825	8.42212E-05	-3.11701	1.67744E-05	0.000153997
Daam2	1.5247725	1.30464E-06	1.923589	2.7417E-07	-3.11547	0.00525229	0.021283
Nav3	0.5548235	0.012280654	1.7713435	0.00006656	-3.11537	0.000961301	0.00506007
Serpina10	4.249114	9.68088E-05	4.15835725	3.08825E-05	-3.10957	5.95983E-08	1.08159E-06
Adamts3	2.56897425	3.81976E-05	2.558071	1.64514E-05	-3.10694	0.000416106	0.0024742
Twist1	1.983587	2.73467E-06	2.3022115	1.10838E-06	-3.09285	0.00122794	0.00624328
AI429214	1.790998	6.17627E-05	1.545031	1.07099E-05	-3.08891	0.00175549	0.00842747
Epha3	3.67248375	7.37153E-05	3.758342	3.54575E-05	-3.07064	7.86686E-06	7.92958E-05
Scara3	4.833925	1.97172E-09	4.06851175	5.30692E-05	-3.06288	3.65125E-10	1.20742E-08
Tmem119	2.07523125	4.28282E-05	2.015285	0.000135748	-3.05683	2.17298E-05	0.000192459
Vash2	2.08945575	3.26739E-05	0.91908075	0.002343552	-3.05101	0.0035651	0.0153725
AI607873	0.6722365	0.036854166	2.32111575	0.000178617	-3.04754	1.9713E-10	6.94527E-09
Hpse	5.25132675	5.23569E-10	2.31617025	0.003349496	-3.0409	4.92127E-10	1.5614E-08
Smad6	-0.01007075	0.479058247	1.61346175	0.000167457	-3.03042	0.000453296	0.002661
Il1r2	2.118013	0.001949363	2.14724275	0.002117753	-3.02512	0.000037097	0.000305711
Arsi	2.401316	0.000247579	2.4421215	9.45146E-05	-3.01827	0.0151937	0.0512988
Kcne4	4.840268	1.01924E-08	5.39005175	0.000001264	-3.0106	2.84672E-08	5.62132E-07
Cxcl13	6.07428575	1.05882E-05	5.32042325	0.000158723	-2.97303	3.01456E-05	0.000255274
Mei4	2.87210325	6.31536E-08	2.80684075	1.41919E-07	-2.96707	0.00468605	0.0193143
Mfap2	2.48788975	3.38363E-05	1.05548075	0.002121235	-2.96088	5.78853E-05	0.000448838
Tnfaip6	5.84717575	2.83943E-06	5.13861675	6.88754E-06	-2.96059	9.77118E-07	1.25875E-05
Ptgir	3.21593175	2.25543E-06	3.00985225	3.3948E-08	-2.95605	2.52281E-08	5.0539E-07
Csf2rb	0.751355	2.70769E-06	2.82981875	0.000194251	-2.93709	1.49256E-06	1.83497E-05
Rcn3	4.42203375	2.55789E-08	1.044605	0.000256046	-2.91832	9.83067E-10	2.87286E-08
Fam26e	3.053677	8.30642E-05	3.311848	5.03845E-06	-2.91364	0.000208706	0.0013694
Arsj	1.71120825	4.39875E-05	1.5367505	7.01617E-05	-2.91029	0.00537814	0.0216904
Fkbp14	1.708814	8.66395E-06	1.26991575	5.60246E-05	-2.90278	0.000570374	0.00324048
Loxl3	2.30181525	2.24092E-05	0.97400275	0.000368465	-2.89064	0.000465808	0.00272274
Fam171b	1.935963	0.00048209	0.7887435	0.017864593	-2.88346	0.00432761	0.0180814
Mmp9	3.30349075	9.57367E-05	-0.815507001	0.022952583	-2.88111	2.26982E-08	4.60044E-07
Glis3	1.6355375	4.38438E-05	-1.314475748	0.000350386	-2.87994	0.00188124	0.00894523
Atoh8	0.645155	0.007492265	1.998114	2.83229E-05	-2.87616	0.00193301	0.00914958
Pla2g15	2.43854025	7.68055E-07	2.084937	2.34684E-06	-2.86184	7.74589E-10	2.32086E-08
Sat2	1.861344	2.03955E-05	1.37932575	2.69513E-05	-2.85262	0.0309349	0.0917209
Mapk8ip1	1.93252775	0.000254659	1.40417975	0.000193716	-2.84614	0.00403448	0.0170525
Far2	2.30867025	0.000045787	2.06799675	5.86456E-07	-2.84438	0.263205	0.470166
Rspo1	1.60504125	0.000819303	1.72353425	0.00114809	-2.81926	0.000216359	0.00141297
Tmem53	1.78727425	8.85003E-06	1.31825475	9.48154E-05	-2.81411	0.000897656	0.00476864
Sult5a1	1.539596	5.98472E-05	1.83953475	4.17859E-05	-2.80905	0.00188433	0.00895683
Coro2b	-0.165522499	0.139882723	1.687568	5.34438E-06	-2.7949	0.00907725	0.0335187
Nsg1	1.8037065	2.75082E-06	-0.289018	0.146155575	-2.77494	9.96899E-06	9.74986E-05
Pi15	5.774578	5.02183E-06	5.683747	4.00966E-06	-2.7708	2.97841E-06	3.38938E-05
Leprel2	2.68149575	2.57891E-05	1.1405565	0.000358421	-2.75563	1.23449E-07	2.05178E-06
Twist2	2.6487425	3.43763E-07	2.96476775	2.20713E-07	-2.745	0.00965011	0.0352437
Aldh3a1	2.81084	8.03447E-05	2.858555	2.00988E-05	-2.74165	0.000619225	0.00346657
Hoxc5	1.6415405	8.84528E-06	1.85490475	1.02121E-05	-2.73639	0.0416048	0.116172
Ecm2	1.63488775	0.000374186	0.98728575	0.003857397	-2.73245	0.00214243	0.00996773
Rsph9	1.725827	0.000220582	1.66674225	0.000142161	-2.73127	0.00399471	0.0169171
Mmp23	4.06079525	4.04348E-07	3.85956175	1.24273E-07	-2.72602	7.13441E-08	1.27029E-06
Lrrc17	4.5034695	1.04333E-06	4.66556475	2.76232E-06	-2.71355	0.000136589	0.000946855
Adra2a	1.64845125	0.004164247	1.6960925	0.003284775	-2.7126	0.000567953	0.00322776
Ly6k	1.48200425	0.000627248	1.6989105	0.000557532	-2.70096	2.10656E-06	2.49727E-05
Sfrp2	5.20796775	6.03731E-08	5.14191275	2.46189E-08	-2.68486	8.61523E-06	8.57141E-05
Btc	2.06547675	0.000201802	2.00679375	0.000362268	-2.67553	0.00872234	0.0324044
Thbs3	2.66649475	1.29079E-05	2.442121	6.34768E-06	-2.67247	0.000618081	0.00346219
Fbn2	2.50691225	2.98767E-08	2.69667925	2.82776E-08	-2.66851	0.0107221	0.0383972
Figf	3.87949525	1.12852E-07	4.0122395	8.17931E-08	-2.60911	0.000452001	0.00265565
Efemp2	3.507792249	7.16555E-08	1.959329	1.18371E-05	-2.60518	3.32153E-07	4.86016E-06
Kirrel3	1.77710925	8.72516E-05	2.87574825	6.51209E-05	-2.55947	0.00473976	0.0195027
Steap3	2.1356245	6.20487E-05	2.31760025	7.6787E-06	-2.55928	3.50085E-05	0.000290346
Wnt5a	4.198213	5.89765E-06	4.0390955	1.09563E-05	-2.52912	0.0456357	0.124921
Vit	2.82897575	7.66019E-06	2.98957075	4.78277E-06	-2.5223	0.00813958	0.0305957
Trpc1	2.302376	0.000144547	2.049509	0.000566494	-2.48206	0.0134119	0.0461431
Srpx	5.27690525	3.2383E-09	5.71606625	1.3638E-09	-2.46208	1.41465E-07	2.31618E-06

Slamf8	2.18943875	3.28582E-06	4.4915455	4.49263E-08	-2.4554	3.50586E-06	3.89344E-05
Fgf7	4.5150615	0.000166566	4.675599	0.000135146	-2.41966	0.000458553	0.00268577
Sfrp4	2.815503	2.37901E-05	2.69992725	0.000163407	-2.41375	0.000161155	0.0010959
Kcnd1	1.23565775	2.61376E-05	1.58920625	7.76472E-07	-2.40928	0.036706	0.105039
Eno2	2.27431125	5.04791E-05	2.2039905	1.36033E-09	-2.38537	0.0025367	0.0115077
Niacr1	2.0587455	3.52686E-06	2.06164975	5.66807E-06	-2.35278	6.95803E-07	9.2986E-06
Bmper	1.61324675	0.001007848	2.10912175	0.000403617	-2.34113	0.000147441	0.00101126
Chl1	2.644633	2.18233E-06	2.628397	1.11071E-06	-2.33143	1.25947E-05	0.000119981
Gpr124	1.7949335	1.27613E-06	-0.29578725	0.009424405	-2.26208	0.00166107	0.00804594
Olfml2b	3.534448	9.70383E-07	4.14247675	7.74862E-06	-2.25557	3.28717E-05	0.000274935
Adam12	3.16671325	1.83659E-06	2.167324	3.20071E-05	-2.24375	2.21644E-05	0.000195681
Rgs17	2.3857765	0.000245672	2.4386695	0.000289571	-2.22252	0.326006	1
Gpr88	2.85013925	3.38897E-08	2.7403465	8.59648E-06	-2.22078	9.09898E-05	0.000663939
Wdr35	2.82048675	8.91548E-08	2.16494025	9.33109E-06	-2.20718	0.000368237	0.00222304
Eya2	2.5259915	8.21612E-06	2.67755825	1.5918E-06	-2.16495	0.00026389	0.00167222
Has1	1.65624125	0.00020777	1.8293655	0.000205867	-2.16196	0.0113966	0.0403616
Nqo1	1.8437775	0.000108288	-0.7176745	0.008335199	-2.14787	0.0115671	0.0408434
Syt13	1.99998175	1.37195E-07	2.301432	6.09237E-07	-2.10142	0.00165369	0.00801769
Il1rl2	4.17692025	1.49334E-05	4.9447555	1.70297E-06	-2.06909	5.40555E-05	0.000423738
Tmem45a	3.21737425	0.000204396	2.9902105	3.15948E-05	-1.99222	0.029637	0.0885183
Xpnpep2	2.26148725	1.18594E-05	2.329059	0.000125441	-1.96538	0.0230133	0.0721216
Dse	4.03064025	6.60881E-05	1.46983525	0.000618074	-1.94985	9.99565E-06	9.77363E-05
Gm11128	2.34419975	0.000596236	2.48387625	0.000339533	-1.91552	0.23391	1
Timd4	3.010533	2.42663E-06	2.346013	8.96863E-06	-1.8995	0.00771099	0.0292793
Leprel1	1.17541775	0.000100757	3.328006499	6.69416E-06	-1.88334	0.00574787	0.022934
Spon2	2.4829125	5.63817E-07	2.6236025	7.11825E-07	-1.7906	0.0106537	0.0382069
Grik5	1.70011125	0.000089661	1.03469375	0.002411399	-1.78225	0.0431873	0.119656
Icosl	3.04766375	2.11006E-05	2.97178925	4.15512E-05	-1.76922	0.00025089	0.00160183
Gpx7	1.251042	3.15121E-05	1.62595375	8.22756E-06	-1.76611	0.00828365	0.0310612
Mrgprf	2.48062725	3.09223E-07	2.53637275	8.55437E-05	-1.70089	0.3811	1
Loxl2	2.6542945	6.00642E-05	-0.683471249	0.008248212	-1.69674	0.00473802	0.0194975
Scg3	3.7736975	0.000254055	4.3782565	6.46338E-05	-1.6095	0.493144	0.70175
Cd248	1.63530275	0.000320849	1.9264745	0.000746745	-1.59701	0.00203987	0.00957001
Il6	1.93292475	0.000789963	4.163473	4.78693E-05	-1.54696	0.0119263	0.0419112
Vcan	1.34441925	0.000183003	1.824867	2.92421E-05	-1.53294	0.0467617	0.127247
Tpbg	1.70051675	6.59994E-05	1.7300505	7.97445E-05	-1.49592	0.0171714	0.0567697
Ripply3	-0.102163001	0.128848465	2.035849	9.52194E-05	-1.41042	0.0403171	0.113255
Pygm	2.128031	8.36233E-06	2.702329	5.05482E-06	-1.25735	0.129074	0.279355
Ccl7	5.75890475	1.0453E-06	4.19478075	5.76788E-07	-1.23413	0.0180877	0.0592045
Phkg1	2.084106	3.56918E-05	2.08713775	5.36531E-06	-1.21612	0.148941	0.311487
Sfmbt2	1.745151	0.000162831	0.17237775	0.175585423	-1.20247	0.408504	0.628239
Dzip1	2.81496675	6.75213E-05	2.8747295	7.18133E-05	-1.19011	0.0635025	0.16234
Spry2	1.86696925	9.83518E-07	1.8979115	8.77169E-07	-1.18142	0.0669423	0.169245
Ccl2	5.053932	7.76295E-06	3.5348185	1.42823E-05	-1.1784	0.0215149	0.0683139
Arhgap10	1.72767325	3.29342E-05	2.59776275	5.62528E-06	-1.17258	0.0487898	0.131437
Sostdc1	4.037506	3.58029E-07	4.05029325	6.80997E-07	-1.1105	0.0167703	0.0556567
Cyp2c44	1.81439975	0.000883866	2.09032975	0.000902302	-1.07806	0.593342	1
Ephb6	2.68273475	0.000171213	2.5702165	2.17501E-05	-1.04898	0.0276089	0.0835255
Chpf	1.8882905	1.07185E-06	2.0461285	5.89038E-07	-0.911664	0.0222142	0.070057
Cdon	3.438021	2.01791E-06	2.8227585	4.55007E-06	-0.859206	0.184985	0.366558
Has2	3.723334749	0.000188048	3.3408715	0.000157063	-0.807563	0.317251	0.532987
Usp11	1.72622975	4.43141E-06	1.118381	0.000223782	-0.806422	0.0726484	0.180189
Adamts10	1.7068575	0.00026207	1.5249015	8.79603E-05	-0.764601	0.0628282	0.160934
Cxcl10	1.69512225	9.05996E-07	0.7510175	0.000117008	-0.650612	0.123195	0.270142
Cd80	1.61504075	0.000101426	1.58034575	8.22447E-08	-0.624978	0.388899	0.609688
Gstm4	2.23682275	7.7836E-06	1.65626475	5.58311E-05	-0.526706	0.467134	0.680384
Gpc3	4.13945225	0.000242509	4.176529	0.000145248	-0.517339	0.366464	0.586535
Cercam	1.72906	3.14476E-07	1.41232	2.89138E-06	-0.481333	0.41299	0.632464
Ncald	2.00474075	1.09948E-06	1.783222	9.83644E-07	-0.408437	0.421073	0.640373
Mboat1	1.2171895	0.000155193	1.6702345	1.98927E-05	-0.396327	0.418041	0.637398
H2-Ob	0.708601	0.004210888	2.135271	1.99502E-05	-0.372153	0.721113	0.866816
Aif1	1.74698275	0.00013342	0.95994925	0.000492559	-0.348979	0.66032	0.828507
Slc15a3	2.44598225	4.43687E-06	3.52302925	6.48595E-07	-0.24279	0.60629	0.790142
Limk1	1.65885625	0.000101843	1.2357695	0.000391762	-0.165547	0.757016	0.887956
Wfdc1	1.81659575	0.000010913	2.20300325	3.35567E-05	-0.149601	0.906145	0.964997
Clec11a	2.69832925	5.81975E-07	2.70926725	3.47276E-06	-0.134048	0.862644	0.944728
Arc	-0.391367	0.088556225	1.887285	4.56915E-06	-0.0692921	0.940615	0.980278
Tnfsf11	1.5432115	0.000918732	1.81286225	0.000161116	0.0938916	0.823418	0.9243
Gli1	1.5916675	4.39338E-06	1.61324125	1.51605E-06	0.17306	0.962846	1
Apbb1ip	2.063814	1.56966E-05	2.18207075	4.94823E-06	0.282524	0.519474	0.724548
Tnfrsf1b	0.380856251	0.03135234	2.164838	6.04638E-05	0.354431	0.411113	0.630871
Cxcl5	2.97378375	0.000089246	3.01998875	0.000120681	0.385455	0.509522	0.715577
Brsk1	1.62431	1.30967E-07	1.41375825	2.02819E-07	0.460147	0.863785	1
Cd72	3.537611499	3.06305E-07	3.7026915	1.14361E-06	0.528591	0.324659	0.541235
Abcb1b	1.81367225	0.000247083	2.90382425	1.86467E-08	0.533219	0.181165	0.361041
Lif	3.467224	0.00007976	1.9115925	0.000847414	0.603026	0.260882	0.467472
Flt3l	3.3064265	1.29876E-06	2.742603	6.54035E-07	0.663704	0.261771	0.468641
Ikbke	1.9973165	5.13923E-05	0.4772235	0.002848309	0.756747	0.0456249	0.124899
Pipox	1.53151575	0.000306688	1.6584045	4.70712E-05	0.785226	0.431198	0.649578
Slc9a9	1.9706655	5.56355E-06	-3.494487748	1.57059E-07	0.811116	0.128537	0.27857
Syt11	1.81590125	2.79764E-05	2.443987	6.87921E-07	0.927649	0.0433411	0.120013
Adora2a	2.35543125	1.22006E-05	3.009279	8.78466E-06	1.08543	0.00666705	0.0260084
Tnfsf8	3.210607	1.5546E-06	2.925725	1.83451E-05	1.16233	0.0030308	0.0133718
Rrad	1.20798875	8.36615E-05	2.080353	1.04655E-06	1.19808	0.00576848	0.0230073
Tnfsf9	1.90833925	1.18727E-05	2.2074	6.1427E-06	1.47981	0.00126382	0.00639602
Fhl2	2.13357	0.001173919	2.439898	0.000231848	2.08012	0.00273452	0.0122576
Lag3	2.9729735	4.896E-07	3.15399725	4.97845E-08	2.29349	0.00143965	0.0071317
Dusp10	2.42601175	0.000183515	1.77649225	6.16172E-05	2.34506	0.000207936	0.00136543

Tmem132c	2.19570975	0.000342903	1.35493325	0.001449526	*low expression values*		
Cubn	1.7846295	0.000161371	1.810778	2.89803E-05	*low expression values/not enough alignments for testing*		
Slco1c1	2.518989	0.000235017	2.71584575	0.000061462	*low expression values/not enough alignments for testing*		
Agtr2	3.8837835	0.000114122	3.992054	1.51814E-05	*low expression values/not enough alignments for testing*		
Ugt3a1	2.12820975	8.14208E-05	2.09151475	7.73954E-05	*low expression values/not enough alignments for testing*		
Gm1987	6.27245175	4.42788E-09	4.9260205	0.000107076	*test status fail*		
Ccl21a	0.414585	0.004135942	3.27120975	0.00080401	*too many fragments in locus*		
